# Decomposition of Copper Formate Clusters: Insight into Elementary Steps of Calcination and Carbon Dioxide Activation

**DOI:** 10.1002/open.201900282

**Published:** 2019-12-17

**Authors:** Tobias F. Pascher, Milan Ončák, Christian van der Linde, Martin K. Beyer

**Affiliations:** ^1^ Institut für Ionenphysik und Angewandte Physik Universität Innsbruck Technikerstraße 25 6020 Innsbruck Austria

**Keywords:** reaction mechanisms, calcination process, mass spectrometry, copper hydrides, decarboxylation

## Abstract

The decomposition of copper formate clusters is investigated in the gas phase by infrared multiple photon dissociation of Cu(II)_*n*_(HCO_2_)_2*n*+1_^−^, *n*≤8. In combination with quantum chemical calculations and reactivity measurements using oxygen, elementary steps of the decomposition of copper formate are characterized, which play a key role during calcination as well as for the function of copper hydride based catalysts. The decomposition of larger clusters (*n* **>**2) takes place exclusively by the sequential loss of neutral copper formate units Cu(II)(HCO_2_)_2_ or Cu(II)_2_(HCO_2_)_4_, leading to clusters with *n*=1 or *n*=2. Only for these small clusters, redox reactions are observed as discussed in detail previously, including the formation of formic acid or loss of hydrogen atoms, leading to a variety of Cu(I) complexes. The stoichiometric monovalent copper formate clusters Cu(I)_*m*_(HCO_2_)_*m*+1_^−^, (*m=*1,2) decompose exclusively by decarboxylation, leading towards copper hydrides in oxidation state +I. Copper oxide centers are obtained via reactions of molecular oxygen with copper hydride centers, species containing carbon dioxide radical anions as ligands or a Cu(0) center. However, stoichiometric copper(I) and copper(II) formate Cu(I)(HCO_2_)_2_^−^ and Cu(II)(HCO_2_)_3_^−^, respectively, is unreactive towards oxygen.

## Graphical abstract

**Calcination and carbon dioxide activation**: Mass spectrometry provides fundamental insight into the complex mechanisms in thermal decomposition of copper formate, with and without the presence of oxygen, which are relevant for the calcination of copper salts and for hydrogen storage applications involving copper hydride‐based catalysts.

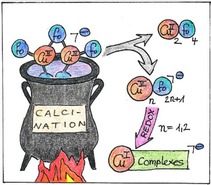

## 1 Introduction

Copper and copper oxide surfaces are widely investigated for their catalytic properties with high selectivity and activity.^1^ They find application in industry, e. g. methanol synthesis,^2^ and environmental technologies like oxidation of carbon monoxide and hydrocarbons^3^ or reduction of nitrogen and sulfur oxides.^4^ Hydride‐based copper catalysts show a very distinct reactivity in organic chemistry and technology.^5^ They are able to selectively decompose formic acid into H_2_ and CO_2._ 6 Furthermore, copper hydride based catalysts may play a key role in hydrogen storage applications.^7^

The calcination of copper salts like copper nitrate or copper formate is a key step in the production of copper‐loaded catalysts.^8^ The catalytic activity strongly depends on the morphology of the catalyst, which in turn is a result of the preparation conditions.^9^ Key parameters are copper salt concentration, which determines the metal loading^10^ as well as the calcination temperature.^11^ Prasad and Singh investigated reactive calcination for different copper salts such as nitrate and acetate.^12^ They found that the catalytic properties of the copper oxide catalyst depend highly on the calcined salt. Acetate showed the highest activity due to a unique morphology.^12^ The calcination process is well understood on a phenomenological level through high‐throughput experiments. However, the molecular processes and atomic level rearrangements are mostly unknown, only the decomposition of some metal salts has been investigated in detail, e. g. metal nitrate.^13^ Understanding the elementary steps during calcination and the reactivity of the products is important for the development of new catalysts and continuous improvement of the existing ones.

The use of atomically defined model systems to describe and identify the elementary steps and reaction mechanisms proved useful.^14^ Copper species received particular attention with respect to carbon dioxide activation. Efficient methanol synthesis was demonstrated on size‐selected copper clusters deposited on aluminum oxide films.^15^ In the gas phase, complexes of copper anions with CO_2_ were investigated by photoelectron and infrared spectroscopy.^16^ Copper hydride anions show reactivity towards CO_2_, leading to formate formation.^17^, ^18^, ^19^ Hydrated Cu^2+^ clusters Cu^2+^(H_2_O)_*n*_ undergo the charge separation reaction^20^ CuOH^+^(H_2_O)_*m*_+H_3_O^+^(H_2_O) at a critical size^21^ of *n*=6, which is higher for copper than for most transition metals.^22^ In the reaction of hydrated Cu^+^ with gaseous HCl, a Cu(I)Cl molecule precipitates in the water cluster, analogous to the precipitation of AgCl.^23^ Reliable thermochemical data are available for the hydration of Cu^+^ and CuOH^+^ from the Armentrout laboratory.^24^ Special attention received the CuO^+^ species in the gas phase. Due to its low bond dissociation energy,^25^ it is a very potent oxidant. In the reaction with methane, H atom abstraction competes with methanol formation, which is in fact the dominant reaction channel.^26^

Recently, we addressed the thermal decomposition of copper formate anions Cu(II)_*n*_(HCO_2_)_2*n*+1_^−^, *n*=1,2.^19^ We showed that the production of formic acid happens via a hydride transfer from formate towards copper, followed by a PCET as the key step in the decomposition. The size of the cluster and oxidation state of the copper(II) centers is crucial for the reaction. Copper formate‐hydride mixtures are obtained from these clusters.^19^ In the present work, we go a step further and model the mechanistic processes during the thermal decomposition for larger and smaller clusters. In addition, we investigate the reactivity of copper formate and its decomposition products with molecular oxygen. These processes play a role during the calcination process and provide mechanistic insights into elementary steps occurring at the surface of copper hydride‐based catalysts. We investigate gas‐phase copper formate clusters Cu(II)_*n*_(HCO_2_)_2*n*+1_^−^ (*n*≤8) using techniques of mass spectrometry. Thermal decomposition during calcination is simulated via infrared multiple photon dissociation (IRMPD) by exciting antisymmetric C−O stretching vibrations. Lastly, O_2_ collisions with heated clusters and decomposition intermediates are investigated to model calcination under the supply of oxygen. In combination with quantum chemical calculations, we describe characteristic processes in the calcination of copper formate on a molecular level.

## Experimental and Theoretical Methods

Anionic copper(II) formate clusters containing isotopically enriched ^63^Cu are introduced into the gas phase through electrospray ionization (ESI), as discussed in detail before.^19^ They are mass selected and trapped in a Bruker APEX Qe 9.4 Tesla Fourier‐transform ion cyclotron resonance (FT‐ICR) mass spectrometer^27^ that is described in more detail elsewhere.^28^ Heating during calcination is simulated via vibrational excitation of asymmetric C−O stretching vibrations, with tunable IR light provided by an EKSPLA NT273‐XIR optical parametric oscillator.^29^ The change in the mass to charge ratio upon decomposition induced by Infrared Multiple Photon Dissociation (IRMPD) is measured and followed as a function of time. Using Collision Induced Dissociation (CID) in addition to ESI, reaction intermediates like Cu(I)_*m*_(HCO_2_)_*m*+1_^−^ (*m*=1, 2) were produced and investigated individually. Oxygen gas is introduced via a pulsed valve into the ICR cell with pressures up to 2.0 ⋅ 10^−7^ mbar. After waiting for five additional seconds for reactions and pump‐down, the mass spectra are acquired.

For the modelling of copper formate clusters, density functional theory (DFT) is employed. We used the B3LYP/def2TZVP and BMK/def2TZVP approaches for smaller copper formate clusters, based on our previous benchmarking.^19^ To model larger clusters with more than four copper atoms, the less computationally demanding DF‐PBE/6‐31+g* theory level was used. The wave function shows instabilities in several cases, its stabilization was carried out for all calculations. Local minimum/transition state character of all stationary points was confirmed by calculation of vibrational frequencies. The nature of transition states was verified through intrinsic reaction coordinate (IRC) calculations or by applying a minor offset along the normal vector of the corresponding imaginary frequency in the transition state followed with steepest decent optimization. Charge analysis was performed using the CHarges from ELectrostatic Potentials using a Grid based method (CHELPG) scheme^30^ with the copper radius of 1.4 Å along with orbital analysis. All calculations were carried out in *Gaussian 09*,^31^ reported energies are zero‐point corrected.

## Results and Discussion

### Thermal Decomposition of Cu(II)_n_(HCO_2_)_2n+1_^−^

**Figure 1:**
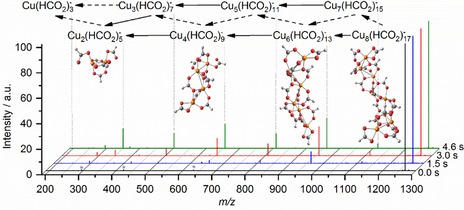
Decomposition of Cu(II)_8_(HCO_2_)_17_^−^ towards Cu(II)(HCO_2_)_3_^−^ irradiated at 1631 cm^−1^. Mass spectra are shown for four representative irradiation times. Possible structures for Cu(II)_*n*_(HCO_2_)_2*n*+1_^−^ (*n*=2,4,6,8) are optimized at the DF‐PBE/6‐31+g* level of theory. Solid arrows indicate predominant fragmentation channels, dashed arrows minor channels. Harmonics of the Fourier transformation corresponding to Cu(II)_8_(HCO_2_)_17_^−^ along with an instrumental noise peak at 297 *m/z* are marked with *.

We start with investigation of dissociation patterns in Cu(II)_*n*_(HCO_2_)_2*n*+1_^−^ (*n*=3,7,8) clusters. The mass spectrum for *n*=8 is shown in Figure 1 while spectra for *n*=3,7 can be found in the SI. In the sequential fragmentation from Cu(II)_8_(HCO_2_)_17_^−^ towards Cu(II)_2_(HCO_2_)_5_^−^ and Cu(II)(HCO_2_)_3_^−^, stoichiometric dicopper tetraformate molecules Cu(II)_2_(HCO_2_)_4_ are lost preferentially, reaction (1), but loss of copper diformate Cu(II)(HCO_2_)_2_ is also observed, reaction (2). The latter is the dominant decomposition channel for Cu(II)_3_(HCO_2_)_7_^−^.

(1) $$\mathrm{Cu}\left(\mathrm{II}\right){}_{n}\left(\mathrm{HCO}{}_{2}\right){}_{2n+1}{}^{-}\to \mathrm{Cu}\left(\mathrm{II}\right){}_{n\text{-}2}\left(\mathrm{HCO}{}_{2}\right){}_{2n\text{-}3}{}^{-}+\mathrm{Cu}\left(\mathrm{II}\right){}_{2}\left(\mathrm{HCO}{}_{2}\right){}_{4}$$

(2) $$\mathrm{Cu}\left(\mathrm{II}\right){}_{n}\left(\mathrm{HCO}{}_{2}\right){}_{2n+1}{}^{-}\to \mathrm{Cu}\left(\mathrm{II}\right){}_{n\text{-}1}\left(\mathrm{HCO}{}_{2}\right){}_{2n\text{-}1}{}^{-}+\mathrm{Cu}\left(\mathrm{II}\right)\;\left(\mathrm{HCO}{}_{2}\right){}_{2}$$

This pattern is consistent with the calculated structures of copper formate clusters. Our calculations suggest that Cu(II)_8_(HCO_2_)_17_^−^ features a chain of copper centers bridged by formate ligands. Additional formate units are attached to the end of the copper chain, exhibiting bidentate or monodentate binding motifs. From the calculated structures, the evaporation of Cu(II)_2_(HCO_2_)_4_ or Cu(II)(HCO_2_)_2_ as the simplest dissociation channel can be expected. In the copper chain of Cu(II)_*n*_(HCO_2_)_2*n*+1_^−^, the distance between Cu ions is ∼3.3–3.6 Å (B3LYP/def2TZVP level of theory, *n*=4), more than 1 Å longer than the bond lengths of 2.22 Å and 2.35 Å in Cu_2_ or Cu_2_^−^, respectively.^32^ This indicates that Cu−Cu interaction in these bridged complexes is weak.

The binding energy of the compact Cu(II)_2_(HCO_2_)_4_ structure compared to two Cu(II)(HCO_2_)_2_ units, reaction (3), is calculated to be Δ*E=*1.30 eV (B3LYP/def2TZVP). Interestingly, the competing Cu(II)_4_(HCO_2_)_9_^−^ dissociation reactions (4a) and (4b) are almost isoenergetic, with slight favor towards the evaporation of dicopper tetraformate. This explains the preferential loss of Cu(II)_2_(HCO_2_)_4_ from large Cu(II)_*n*_(HCO_2_)_2n+1_^−^ clusters. Only when *n*=3 is reached, the evaporation of one neutral Cu(II)(HCO_2_)_2_ unit towards Cu(II)_2_(HCO_2_)_5_^−^, reaction (5a) below, is favored compared to the evaporation of Cu(II)_2_(HCO_2_)_4_, reaction (5b). During the evaporation of stoichiometric copper formate molecules, the formal oxidation state +II of each copper center is preserved. A similar dissociation pattern was recorded for Cu(II)_7_(HCO_2_)_15_^−^, see SI (Figure S1), where again Cu(II)_2_(HCO_2_)_4_ is lost preferentially until *n*=3 is reached.

(3) $$\mathrm{Cu}\left(\mathrm{II}\right){}_{2}\left(\mathrm{HCO}{}_{2}\right){}_{4}\to 2\mathrm{Cu}\left(\mathrm{II}\right)\left(\mathrm{HCO}{}_{2}\right){}_{2}\;\;\Delta E=1.30\;\mathrm{eV}$$

(4a) $$\mathrm{Cu}\left(\mathrm{II}\right){}_{4}\left(\mathrm{HCO}{}_{2}\right){}_{9}{}^{-}\to \mathrm{Cu}\left(\mathrm{II}\right){}_{2}\left(\mathrm{HCO}{}_{2}\right){}_{5}{}^{-}+\mathrm{Cu}\left(\mathrm{II}\right){}_{2}\left(\mathrm{HCO}{}_{2}\right){}_{4}\;\;\Delta E=0.91\;\mathrm{eV}$$

(4b) $$\mathrm{Cu}\left(\mathrm{II}\right){}_{4}\left(\mathrm{HCO}{}_{2}\right){}_{9}{}^{-}\to \mathrm{Cu}\left(\mathrm{II}\right){}_{3}\left(\mathrm{HCO}{}_{2}\right){}_{7}{}^{-}+\mathrm{Cu}\left(\mathrm{II}\right)\left(\mathrm{HCO}{}_{2}\right){}_{2}\;\;\Delta E=1.11\;\mathrm{eV}$$

(5a) $$\mathrm{Cu}\left(\mathrm{II}\right){}_{3}\left(\mathrm{HCO}{}_{2}\right){}_{7}{}^{-}\to \mathrm{Cu}\left(\mathrm{II}\right){}_{2}\left(\mathrm{HCO}{}_{2}\right){}_{5}{}^{-}+\mathrm{Cu}\left(\mathrm{II}\right)\left(\mathrm{HCO}{}_{2}\right){}_{2}\;\;\Delta E=1.10\;\mathrm{eV}$$

(5b) $$\mathrm{Cu}\left(\mathrm{II}\right){}_{3}\left(\mathrm{HCO}{}_{2}\right){}_{7}{}^{-}\to \mathrm{Cu}\left(\mathrm{II}\right)\left(\mathrm{HCO}{}_{2}\right){}_{3}{}^{-}+\mathrm{Cu}\left(\mathrm{II}\right){}_{2}\left(\mathrm{HCO}{}_{2}\right){}_{4}\;\;\Delta E=1.16\;\mathrm{eV}$$

Small Cu(II)_*n*_(HCO_2_)_2*n*+1_^−^, *n=*1, 2, clusters exhibit a very different dissociation behavior. We have shown before that for Cu(II)_2_(HCO_2_)_5_^−^, the evaporation of Cu(II)(HCO_2_)_2_ leading to Cu(II)(HCO_2_)_3_^−^ is observed as a minor channel, followed by decarboxylation and hydrogen radical dissociation forming Cu(I)(HCO_2_)_2_^−^. The predominant dissociation channel of Cu(II)_2_(HCO_2_)_5_^−^, however, leads to formation of Cu(I)_2_(HCO_2_)_3_^−^, see Ref. [19] for details. The decomposition mass spectra of the resulting monovalent fragments, Cu(I)_2_(HCO_2_)_3_^−^ and Cu(I)(HCO_2_)_2_^−^, are available in the SI for selected irradiation times. Sequential decarboxylation leading to copper hydrides is observed in both cases.

**Figure 2:**
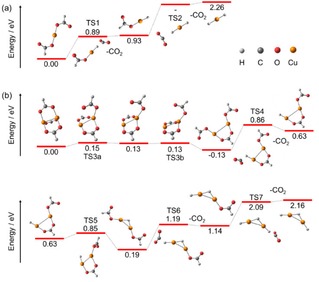
Simplified reaction scheme starting with a) Cu(I)(HCO_2_)_2_^−^ and b) Cu(I)_2_(HCO_2_)_3_^−^ leading to a) Cu(I)H_2_^−^ and b) Cu(I)_2_H_3_^−^, respectively, calculated at the B3LYP/def2TZVP level of theory. In the case of TS2 and TS3, slightly different potential energy surfaces are predicted for B3LYP/def2TZVP and BMK/def2TZVP methods, see Figure S6 in SI for details. For TS2, the BMK/def2TZVP structure is shown. Structures in the decomposition pathway from Cu(I)(HCO_2_)_2_^−^ to HCu(I)(HCO_2_)^−^ and from Cu(I)_2_(HCO_2_)_3_^−^ to HCu(I)_2_(HCO_2_)_2_^−^ are in part reproduced from Ref. [19].

**Figure 3:**
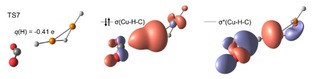
Structure of the hydride transfer transition state TS7 for reaction (6) along with the doubly occupied three‐center σ(Cu−H−C) and unoccupied σ*(Cu−H−C) orbitals and the CHELPG charge of the transferred hydrogen atom. Calculated at the B3LYP/def2TZVP level of theory.

The potential energy surface, starting with Cu(I)(HCO_2_)_2_^−^ and Cu(I)_2_(HCO_2_)_3_^−^, is illustrated in Figure 2. The final decarboxylation products are Cu(I)H_2_^−^ and Cu(I)_2_H_3_^−^. All decarboxylation steps are endothermic. They proceed through hydride transfer from a HCO_2_^−^ ligand to the copper center. In Figure 3, the orbitals participating in the hydride transfer are illustrated for breaking the C−H bond in TS7 within reaction

(6) $$\mathrm{Cu}\left(I\right){}_{2}\left(\mathrm{HCO}{}_{2}\right)H{}_{2}{}^{-}\to \mathrm{Cu}\left(I\right){}_{2}H{}_{3}{}^{-}+\mathrm{CO}{}_{2}\;\;\Delta E=1.02\;\mathrm{eV}$$

while orbitals for other TSs are shown in the SI (Figure S9). Orbital analysis of the TSs confirms a doubly occupied three‐center σ‐bond across the C−H−Cu moiety and an unoccupied antibonding three‐center sigma orbital for all cases. Such a situation is typical for a hydride transfer^33^ along with the fact that the C−H−Cu atoms are not oriented linearly in the transition state.^34^ Hydride transfer is also confirmed by the negative partial charge of the hydrogen atom ranging from −0.31 e to −0.44 e and the absence of excess spin density for all decarboxylation reactions of the Cu(I) species.

For the reaction of Cu(I)(HCO_2_)H^−^→Cu(I)H_2_^−^+CO_2_, calculations at the BMK/def2TZVP predict a transition state (TS2) for the hydride transfer breaking the C−H bond, lying with 1.21 eV well below the CO_2_ dissociation limit of Δ*E*=1.38 eV with respect to Cu(I)(HCO_2_)H^−^. However, at the B3LYP/def2TZVP level, the transition state vanishes and the calculated decarboxylation reaction occurs without a barrier. This is in line with the previously reported barrierless CO_2_ activation, which is the reverse reaction of the decarboxylation.^17^ The relatively low rate coefficient for the reverse reaction from copper hydride to copper formate reported by O'Hair and co‐workers^17^ might be explained by this additional TS2 within the BMK method. Also He and co‐workers reported CO_2_ activation by Cu_2_H_2_^−^, with strong evidence for the formation of a C−H bond, leading to Cu_2_(HCO_2_)H^−^.^18^ As all decarboxylation reactions observed here are endothermic, the reverse reactions with copper centers leading to formate seem to be a general feature in the activation of CO_2_ by copper(I) hydrides.

### Reactions Involving O_2_

To investigate potential elementary steps of calcination under heating in oxygen or air, the reactivity of oxygen with copper formate clusters and their heated fragment ions was investigated by introducing O_2_ via a pulsed valve. Cu(II)(HCO_2_)_3_^−^ is selected as a representative parent ion since its thermal decomposition products include all types of observed species after irradiation for 10 s combined with collisional activation. This gives access to different copper oxidation states, namely Cu(I)(HCO_2_)_2_^−^ and Cu(0)(HCO_2_)^−^/HCu(I)(CO_2_)^−^ as well as formate/hydride mixtures, namely Cu(I)H_2_^−^, Cu(I)(HCO_2_)H^−^ and Cu(II)(HCO_2_)_2_H^−^. Particularly interesting are traces of decomposition products containing carbon dioxide radical anions, i. e. Cu(I)(HCO_2_)CO_2_^−^ and HCu(I)(CO_2_)^−^, see Ref. [19] for details on their origin. The latter are expected to be very reactive towards oxygen through a CO_2_ to O_2_ exchange. The experiment with O_2_ may thus allow us to determine whether Cu(0)(HCO_2_)^−^ or HCu(I)CO_2_^−^ is present as decomposition product, since the latter is expected to undergo ligand exchange of CO_2_ against O_2_.

**Figure 4:**
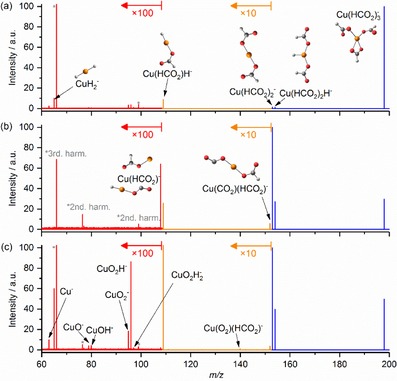
The reactivity of Cu(II)(HCO_2_)_3_^−^ a) after 10 s wait period without laser irradiation followed by pulsing oxygen with 5 s pump down delay; b) with laser irradiation at 1675 cm^−1^ for 10 s forming reactive, heated fragment species before pulsing oxygen; c) with laser irradiation for 10 s followed by pulsing in oxygen with 5 s pump down delay. Calculated structures at the B3LYP/def2TZVP level of theory are shown. Harmonics of the Fourier transformation are marked with *.

Without laser irradiation (Figure 4a), collisions with O_2_ together with BIRD lead to the same decomposition products as IR heating, all the way down to Cu(I)H_2_, albeit in very small amounts. In addition, minute traces of oxide and hydroxide species, namely CuO^−^, CuOH^−^, CuO_2_^−^ and CuO_2_H^−^, as well as Cu^−^ are observed after the O_2_ pulse. The products newly observed in the presence of O_2_ are most likely formed in reactions with reactive fragments like Cu(I)H_2_^−^.

With laser irradiation (Figure 4b), a larger variety of reactive species is formed, like e. g. Cu(I)(HCO_2_)CO_2_^−^. After introducing O_2_, formation of copper oxide, peroxide and hydroxide species like Cu(O_2_)(HCO_2_)^−^, CuO_2_^−^, CuO^−^, CuOH^−^, CuO_2_H^−^, and CuO_2_H_2_^−^, is observed (Figure 4c), as well as bare Cu^−^. We observe simple ligand exchange reactions of CO_2_ against O_2_ with the depletion of the Cu(CO_2_)(HCO_2_)^−^ and HCu(CO_2_)^−^ peaks, resulting in Cu(O_2_)(HCO_2_)^−^ and HCu(O_2_)^−^, respectively. HCu(O_2_)^−^ is most likely formed from HCu(I)(CO_2_)^−^/Cu(0)(HCO_2_)^−^ at *m*/*z* 108. Other fragments, e. g. CuOH^−^ or Cu^−^, require more complex rearrangements. Unfortunately, the signal intensity of the fragments is too low to allow for mass selection followed by reactivity studies.

**Figure 5:**
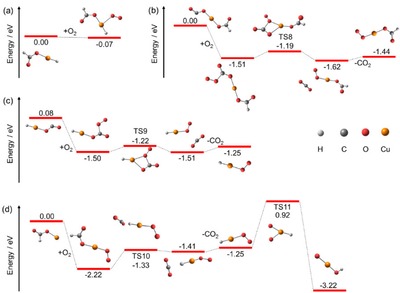
Simplified reaction scheme with oxygen for selected fragments forming upon irradiation of Cu(II)(HCO_2_)_3_^−^. a) Addition of O_2_ to Cu(I)(HCO_2_)H^−^; b) O_2_/CO_2_ exchange on Cu(I)(HCO_2_)CO_2_^−^; c) O_2_/CO_2_ exchange on HCu(I)CO_2_^−^; d) decarboxylation of Cu(0)(HCO_2_)^−^ after O_2_ addition; note that energies in c) and d) share the same zero point in energy. Calculated at the B3LYP/def2TZVP level of theory with zero‐point corrected energies provided in eV. BMK/def2TZVP results are shown for comparison in Figure S7.

Quantum chemical calculations are carried out to understand the low reactivity of the precursor ion and to identify reaction pathways that can explain the observed products. All energies given in the following are calculated at the B3LYP/def2TZVP level including zero‐point correction. In the encounter complexes of the relevant formate/hydride species with oxygen, (O_2_)Cu(II)(HCO_2_)_3_^−^, (O_2_)Cu(II)(HCO_2_)_2_H^−^ and (O_2_)Cu(I)(HCO_2_)_2_^−^, the O_2_ binding energy is below 0.02 eV. For Cu(I)(HCO_2_)H^−^, the O_2_ binding energy at the copper center is as low as 0.07 eV, see Figure 5a. Due to the low binding energies, the encounter complexes are short lived, which is one reason for the low reactivity of copper formate/hydride species towards O_2_.

For the reaction of Cu(I)(HCO_2_)CO_2_^−^ with oxygen illustrated in Figure 5b, the oxygen molecule does not bind to the copper center in the first step. Instead, O_2_ binds to the CO_2_^−^ radical resulting in a CO_4_^−^ ligand. The CO_4_^−^ can then flip towards the copper center through a transition state at Δ*E*=−1.19 eV, allowing the oxygen to interact with the copper with simultaneous evaporation of CO_2_, reaction (7). The exchange reaction is exothermic relative to the separated reactants. Such CO_4_^−^ intermediates play a key role in the CO_2_^−^ to O_2_ exchange for other species and have been discussed and studied before, particularly in water clusters.^35^

(7) $$\mathrm{Cu}\left(I\right)\left(\mathrm{HCO}{}_{2}\right)\mathrm{CO}{}_{2}{}^{-}+O{}_{2}\to \mathrm{Cu}\left(I\right)\left(\mathrm{HCO}{}_{2}\right)\mathrm{CO}{}_{4}{}^{-}\to \mathrm{Cu}\left(I\right)\left(\mathrm{HCO}{}_{2}\right)O{}_{2}{}^{-}+\mathrm{CO}{}_{2}\;\;\Delta E=-1.44\;\mathrm{eV}$$

A very similar reaction mechanism is predicted for HCu(I)CO_2_^−^, see Figure 5c and reaction (8a), leading to a CO_2_ to O_2_ exchange. However, the calculated PES in Figure 5d suggests that an alternative mechanism is possible, starting from the Cu(I)(HCO_2_)^−^ structure, reaction (8b). The copper center in Cu(0)(HCO_2_)^−^ is very reactive towards oxygen. The resulting excess energy is enough to initiate the decarboxylation via TS10, followed by the evaporation of the CO_2_ unit, leading to HCu(I)(O_2_)^−^. Rearrangement to OCu(II)(OH)^−^, reaction (8c), would be very exothermic, but copper insertion into the O_2_ bond requires Δ*E*=0.92 eV with respect to the entrance channel, which renders this pathway inaccessible. The two reaction mechanisms for the formation of HCu(I)(O_2_)^−^, (8a,b), show that the formation is feasible for both Cu(0)(HCO_2_)^−^ and HCu(I)(CO_2_)^−^ of the *m*/*z* 108 precursor, and we cannot assign the structure on this basis.

(8a) $$\mathrm{HCu}\left(I\right)\left(\mathrm{CO}{}_{2}\right){}^{-}+O{}_{2}\to \mathrm{HCu}\left(I\right)\left(\mathrm{CO}{}_{4}\right){}^{-}\to \mathrm{HCu}\left(I\right)\left(O{}_{2}\right){}^{-}+\mathrm{CO}{}_{2}\;\;\Delta E=-1.33\;\mathrm{eV}$$

(8b) $$\mathrm{Cu}\left(0\right)\left(\mathrm{HCO}{}_{2}\right){}^{-}+O{}_{2}\to \mathrm{Cu}\left(I\right)\left(\mathrm{HCO}{}_{2}{}^{-}\right)O{}_{2}\to \mathrm{HCu}\left(I\right)\left(O{}_{2}\right){}^{-}+\mathrm{CO}{}_{2}\;\;\Delta E=-1.25\;\mathrm{eV}$$

(8c) $$\mathrm{Cu}\left(0\right)\left(\mathrm{HCO}{}_{2}\right){}^{-}+O{}_{2}\to \mathrm{Cu}\left(I\right)\left(\mathrm{HCO}{}_{2}{}^{-}\right)O{}_{2}\to \mathrm{OCu}\left(\mathrm{II}\right)\mathrm{OH}{}^{-}+\mathrm{CO}{}_{2}\;\;\Delta E=-3.22\;\mathrm{eV}$$

**Figure 6:**
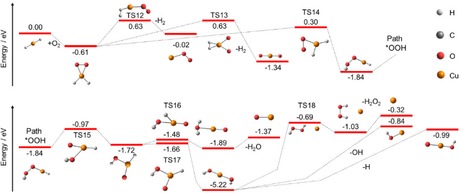
Simplified reaction scheme with oxygen for Cu(I)(H_2_)^−^. Cu(0)O_2_^−^ and OCu(II)O^−^ can be formed under hydrogen dissociation. If one hydrogen is transferred onto the O_2_ unit in the first step (Path *OOH), different pathways open up, leading to the formation of Cu(I)O^−^, Cu(−I)^−^, Cu(0)OH^−^ and OCu(II)OH^−^ under the dissociation of H_2_O, H_2_O_2_, OH and H, respectively. Calculated at the B3LYP/def2TZVP level of theory with zero‐point corrected energies in eV. BMK/def2TZVP results can be found for comparison in Figure S8.

In contrast to the formate species, copper hydride Cu(I)H_2_^−^ binds O_2_ reasonably well, reaction (9) and Figure 6. In agreement with its negligible experimental abundance, however, this ion cannot stabilize in a binary collision. Hydrogen elimination from Cu(II)H_2_(O_2_)^−^ may occur in two ways, leading to the peroxo complex Cu(0)(O_2_)^−^ or the dioxide OCu(II)O^−^, reactions (9a) and (9b), respectively. Both reactions face transition states at Δ*E*=0.63 eV relative to the entrance channel, Figure 6. Formation of the peroxo complex, reaction (9a), is essentially thermoneutral while breaking of the dioxygen bond with formation of OCu(II)O^−^, reaction (9b), is significantly exothermic. Further reactions are initiated by the rearrangement to the hydride‐hydroperoxy species HCu(I)(OOH)^−^, reaction (9c), which faces a barrier of 0.30 eV relative to the entrance channel. All these pathways are accessible in the heated environment.

(9) $$\mathrm{Cu}\left(I\right)H{}_{2}{}^{-}+O{}_{2}\to \mathrm{Cu}\left(\mathrm{II}\right)H{}_{2}\left(O{}_{2}\right){}^{-}\;\;\Delta E=-0.61\;\mathrm{eV}$$

(9a) $$\mathrm{Cu}\left(I\right)H{}_{2}{}^{-}+O{}_{2}\to \mathrm{Cu}\left(\mathrm{II}\right)H{}_{2}\left(O{}_{2}\right){}^{-}\to \mathrm{Cu}\left(0\right)\left(O{}_{2}\right){}^{-}+H{}_{2}\;\;\Delta E=-0.02\;\mathrm{eV}$$

(9b) $$\mathrm{Cu}\left(I\right)H{}_{2}{}^{-}+O{}_{2}\to \mathrm{Cu}\left(\mathrm{II}\right)H{}_{2}\left(O{}_{2}\right){}^{-}\to \mathrm{OCu}\left(\mathrm{II}\right)O{}^{-}+H{}_{2}\;\;\Delta E=-1.34\;\mathrm{eV}$$

(9c) $$\mathrm{Cu}\left(I\right)H{}_{2}{}^{-}+O{}_{2}\to \mathrm{Cu}\left(\mathrm{II}\right)H{}_{2}\left(O{}_{2}\right){}^{-}\to \mathrm{HCu}\left(I\right)\left(\mathrm{OOH}\right){}^{-}\;\;\Delta E=-1.84\;\mathrm{eV}$$

Following reaction (9c), the Cu center inserts into the O−O bond of the hydroperoxy group HOO, which affords elimination of H_2_O and formation of Cu(I)O^−^, reaction (10a). Alternatively, the hydrogen atom can be transferred to the other oxygen atom, leading to the dihydroxy species Cu(I)(OH)_2_^−^, reaction (10a)b):

(10a) $$\mathrm{Cu}\left(I\right)H{}_{2}{}^{-}+O{}_{2}\to \mathrm{HCu}\left(I\right)\left(\mathrm{OOH}\right){}^{-}\to \mathrm{Cu}\left(I\right)O{}^{-}+H{}_{2}O\;\;\Delta E=-1.37\;\mathrm{eV}$$

(10b) $$\mathrm{Cu}\left(I\right)H{}_{2}{}^{-}+O{}_{2}\to \mathrm{HCu}\left(I\right)\left(\mathrm{OOH}\right){}^{-}\to \mathrm{Cu}\left(I\right)\left(\mathrm{OH}\right){}_{2}{}^{-}\;\;\Delta E=-5.22\;\mathrm{eV}$$

(10c) $$\mathrm{Cu}\left(I\right)H{}_{2}{}^{-}+O{}_{2}\to \mathrm{Cu}\left(I\right)\left(\mathrm{OH}\right){}_{2}{}^{-}\to \mathrm{OCu}\left(\mathrm{II}\right)\left(\mathrm{OH}\right){}^{-}+H\;\;\Delta E=-0.99\;\mathrm{eV}$$

(10d) $$\mathrm{Cu}\left(I\right)H{}_{2}{}^{-}+O{}_{2}\to \mathrm{Cu}\left(I\right)\left(\mathrm{OH}\right){}_{2}{}^{-}\to \mathrm{Cu}\left(0\right)\left(\mathrm{OH}\right){}^{-}+\mathrm{OH}\;\;\Delta E=-0.84\;\mathrm{eV}$$

(10e) $$\mathrm{Cu}\left(I\right)H{}_{2}{}^{-}+O{}_{2}\to \mathrm{Cu}\left(I\right)\left(\mathrm{OH}\right){}_{2}{}^{-}\to \mathrm{Cu}{}^{-}+H{}_{2}O{}_{2}\;\;\Delta E=-0.32\;\mathrm{eV}$$

The reaction (10b) is significantly exothermic, which may cause a hydrogen atom or hydroxyl radical to dissociate, forming OCu(II)(OH)^−^, reaction (10c), or Cu(0)(OH)^−^, reaction (10d), respectively. If both hydroxyl groups recombine and dissociate as a hydrogen peroxide molecule in reaction (10e), Cu^−^ is left behind. The transition state for formation of hydrogen peroxide is with Δ*E*=−0.69 eV still well below the entrance channel. The PES shows that the formation of hydroxyl groups on copper centers is very favorable. Cu(I)(OH)_2_^−^ as the global minimum for this system is supported by the experimental observation of a CuO_2_H_2_^−^ peak in the mass spectrum in Figure 4c. The observed peak is very small and barely above noise level, but this is realistic, given the variety of energetically accessible decomposition reactions and the UHV conditions of the FT‐ICR cell. Most ions follow one of the four dissociation channels, i. e. loss of H_2_O_2_, H_2_O, H or OH, leading to the observed ions Cu^−^, Cu(I)O^−^, OCu(II)(OH)^−^ or Cu(0)(OH)^−^, respectively. Each of these channels is overall significantly exothermic.

## Conclusions

To obtain an understanding of elementary steps during copper formate calcination as well as the mechanism of copper hydride based catalysts, we investigated and characterized the molecular processes during the decomposition of copper formate nanoparticles in the gas phase by infrared multiple photon dissociation of Cu(II)_*n*_(HCO_2_)_*2n+*1_^−^, *n*≤8. Large copper formate clusters evaporate small stoichiometric entities Cu(II)_2_(HCO_2_)_4_ and Cu(II)(HCO_2_)_2_ until Cu(II)_2_(HCO_2_)_5_^−^ or Cu(II)(HCO_2_)_3_^−^ is reached. For these clusters, the favorable evaporation of Cu(II)_2_(HCO_2_)_4_ is not possible anymore, and new reaction pathways are observed. The binary complex is predominantly reduced through decarboxylation followed by a proton‐coupled electron transfer, leading to formation of formic acid. The copper triformate complex decomposes by decarboxylation followed by hydrogen radical dissociation. The resulting monovalent copper formate clusters Cu(I)_2_(HCO_2_)_3_^−^ and Cu(I)(HCO_2_)_2_^−^ undergo sequential decarboxylation, leading to copper hydrides Cu(I)_2_H_3_^−^ or Cu(I)H_2_^−^. As these reactions are endothermic, the reverse reactions are promising candidates for the activation of CO_2_. Copper formate Cu(II)(HCO_2_)_3_^−^ and its reduced form Cu(I)(HCO_2_)_2_^−^ are unreactive towards oxygen. However, Cu(0)(HCO_2_)^−^ and the hydride fragment Cu(I)H_2_^−^ showed very exothermic reactions with oxygen, resulting in the formation of copper oxide and hydride mixtures. The complexes featuring a carbon dioxide radical anion ligand, which we observe in small amounts, exchange CO_2_ against O_2_ via an intermediate containing a CO_4_^−^ ligand.

## Conflict of interest

The authors declare no conflict of interest.

## Supporting information

As a service to our authors and readers, this journal provides supporting information supplied by the authors. Such materials are peer reviewed and may be re‐organized for online delivery, but are not copy‐edited or typeset. Technical support issues arising from supporting information (other than missing files) should be addressed to the authors.

Supplementary
