# Supplementary material for: Decomposition of Copper Formate Clusters: Insight into Elementary Steps of Calcination and Carbon Dioxide Activation
Source: ChemistryOpen. 2019 Dec 17;8(12):1453–9. doi: 10.1002/open.201900282 (PMC6916659; doi:10.1002/open.201900282)
Supplement: Supplementary file 1 — Supplementary [file OPEN-8-1453-s001.pdf]

ChemistryOPEN
Open Access

Supporting Information

© Copyright Wiley-VCH Verlag GmbH & Co. KGaA, 69451 Weinheim, 2019

Decomposition of Copper Formate Clusters: Insight into Elementary Steps of Calcination and Carbon Dioxide Activation

Tobias F. Pascher, Milan Ončák, Christian van der Linde, and Martin K. Beyer*© 2019 The Authors. Published by Wiley-VCH Verlag GmbH & Co. KGaA.
This is an open access article under the terms of the Creative Commons Attribution License, which permits use, distribution and reproduction in any medium, provided the original work is properly cited.

Experiments

In Figure S1, the mass spectrum for the decomposition upon irradiation of the asymmetric C-O stretching vibration of copper formate for copper formate anions with an odd number of copper ions is shown (here $Cu(II)_7(HCO_2)_{15}^-$) for selected irradiation times to demonstrate the dominant decomposition via evaporating $Cu(II)_2(HCO_2)_4$ until *n* = 3. In Figures S2–4, the mass spectra for the decomposition of smaller copper formate clusters ($Cu(II)_3(HCO_2)_7^-$, $Cu(I)_2(HCO_2)_3^-$ and $Cu(I)(HCO_2)_2^-$) are shown, which start exhibiting a different behavior upon irradiation of the asymmetric C-O stretching vibration for selected irradiation times.

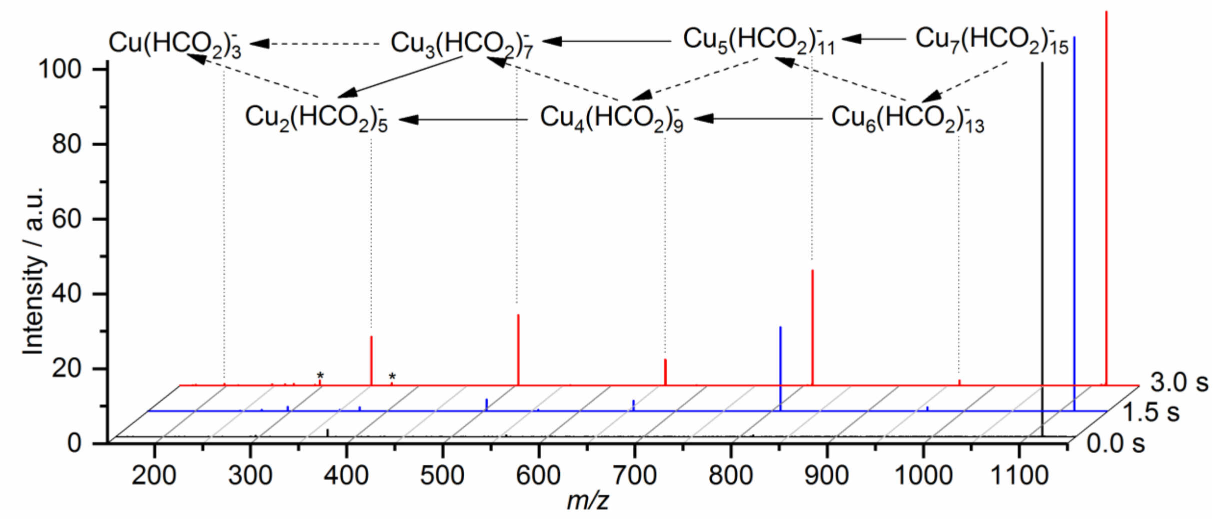

Figure S1. The decomposition of Cu(II)7(HCO2)15- towards Cu(II)(HCO2)3- for irradiation at 1631 cm-1 for three selected irradiation times. The predominant sequential fragmentation channels are illustrated as solid arrows while minor fragmentation pathways are shown as dashed arrows.

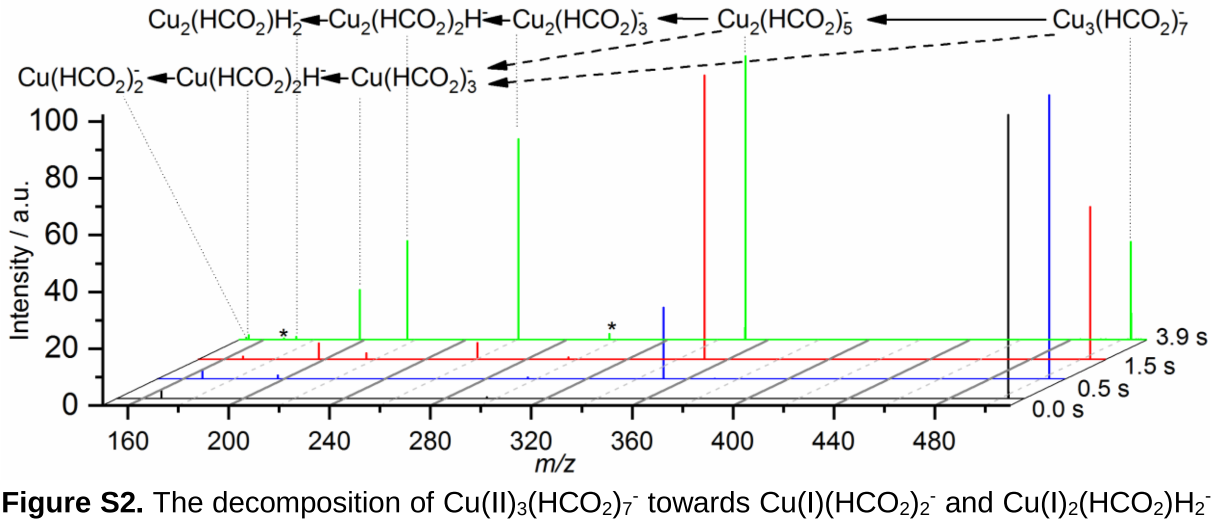

Figure S2. The decomposition of Cu(II)$_3$(HCO$_2$)$_7^-$ towards Cu(I)(HCO$_2$)$_2^-$ and Cu(I)$_2$(HCO$_2$)H$_2^-$ through Cu(II)(HCO$_2$)$_3^-$ or Cu(II)$_2$(HCO$_2$)$_5^-$, respectively, irradiated at 1632 cm^{-1} for four selected irradiation times. The predominant sequential fragmentation channels are illustrated as solid arrows while minor fragmentation pathways are shown as dashed arrows.

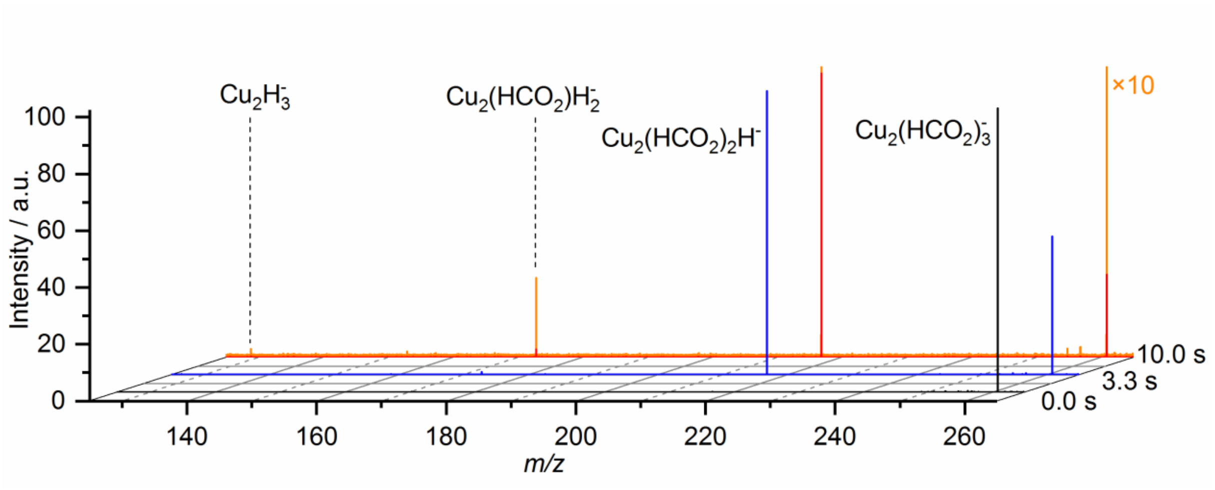

Figure S3. The decomposition of $Cu(I)_2(HCO_2)_3^-$ towards $Cu(I)_2H_3^-$ when irradiated at 1611 cm^{-1} for three selected irradiation times.

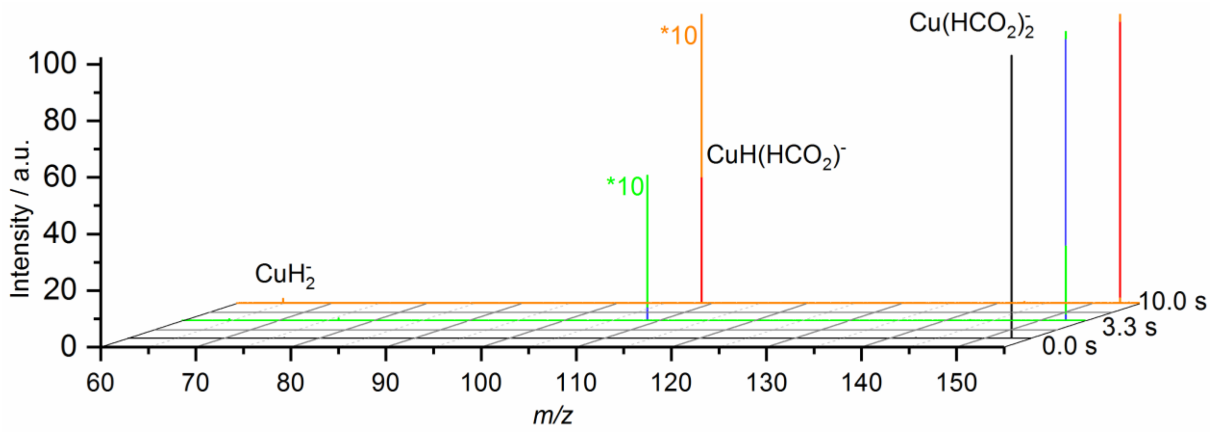

Figure S4. The decomposition of $Cu(I)(HCO_2)_2^-$ towards $Cu(I)H_2^-$ when irradiated at 1643 cm^{-1} for three selected irradiation times.

Theoretical methods and results

For energy benchmarking of basis sets and methods for two reactions observed with copper formate, see Ref. [1]. Based on those results, the B3LYP and BMK DFT functional with the def2TZVP basis set is chosen for geometry optimization as it agrees well with higher-level methods along with a denser integration grid (“Int=UltraFine” in *Gaussian 09*). Structural benchmarking for the PBE functional with density fitting (DF-PBE) and 6-31G* basis set compared to B3LYP/def2TZVP is shown in Figure S5 for $Cu(II)_3(HCO_2)_7^-$ and $Cu(II)_4(HCO_2)_9^-$. While the copper-copper bonding interaction is overestimated with Δ(Cu-Cu) ≈ 0.3 Å compared to using B3LYP/def2TZVP, the other bond lengths are well reproduced with Δ*r* < 0.1 Å and the interactions follow overall the same trend upon increasing cluster size. Therefore, basic geometry information can be gained within the computational limitations even for larger copper formate clusters (e.g. $Cu(II)_8(HCO_2)_{17}^-$) using DF-PBE/6-31G*.

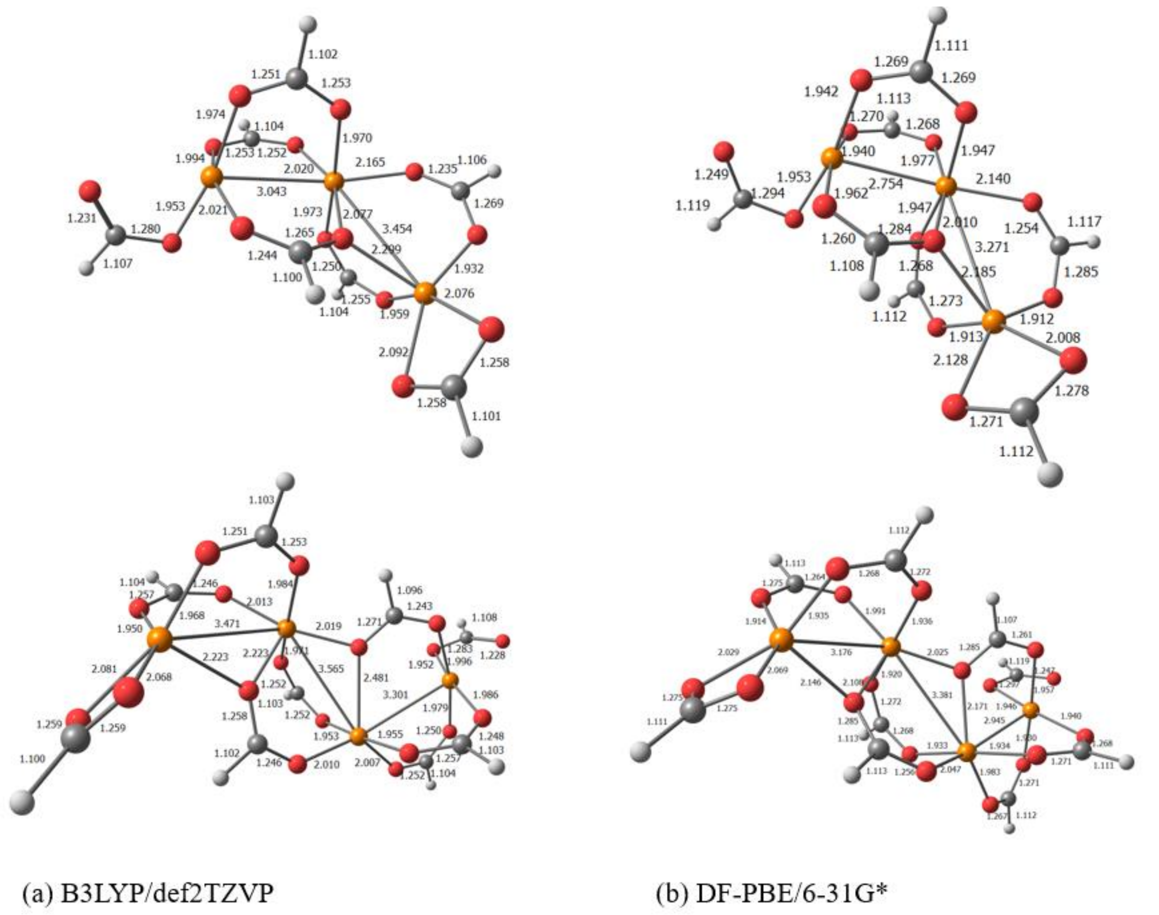

Figure S5. Geometry comparison along with bond lengths in Å of $Cu(II)_3(HCO_2)_7^-$ and $Cu(II)_4(HCO_2)_9^-$ optimized at the a) B3LYP/def2TZVP and b) DF-PBE/6-31G* level.

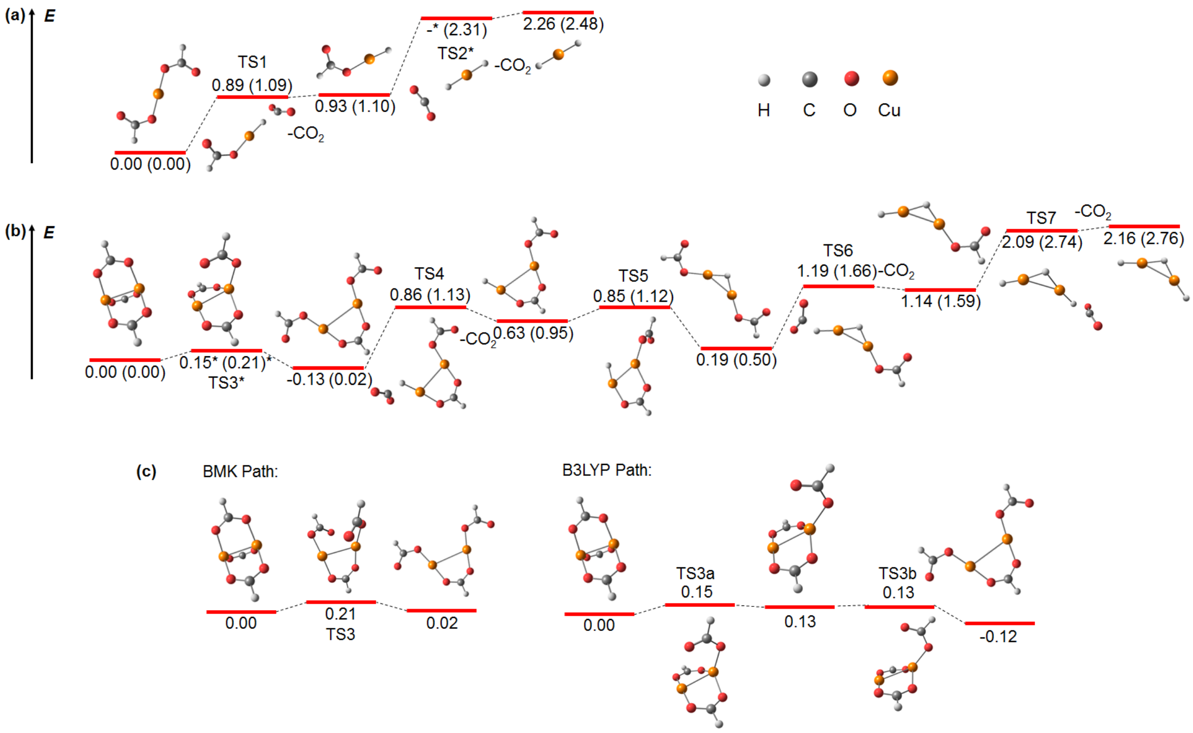

Figure S6. In a) and b), comparison of the potential energy surface in Figure 2 from the DFT method B3LYP/def2TZVP to BMK/def2TZVP in brackets. For TS2 and TS3, slightly different potential energy surfaces are predicted for B3LYP/def2TZVP and BMK/def2TZVP. TS2 only exists within the BMK method while the B3LYP pathway occurs barrierlessly. In c), Pathway on the potential energy surface for the opening of the formate ligands in TS3* calculated at the BMK/def2TZVP and B3LYP/def2TZVP level respectively. While BMK yields one transition state for breaking both Cu-O bonds, the two ligands break them sequentially according the B3LYP pathway. The methods favor different isomers as global minimum for $Cu(I)_2(HCO_2)_3^-$, but the decarboxylation is expected to occur only through the open isomer.

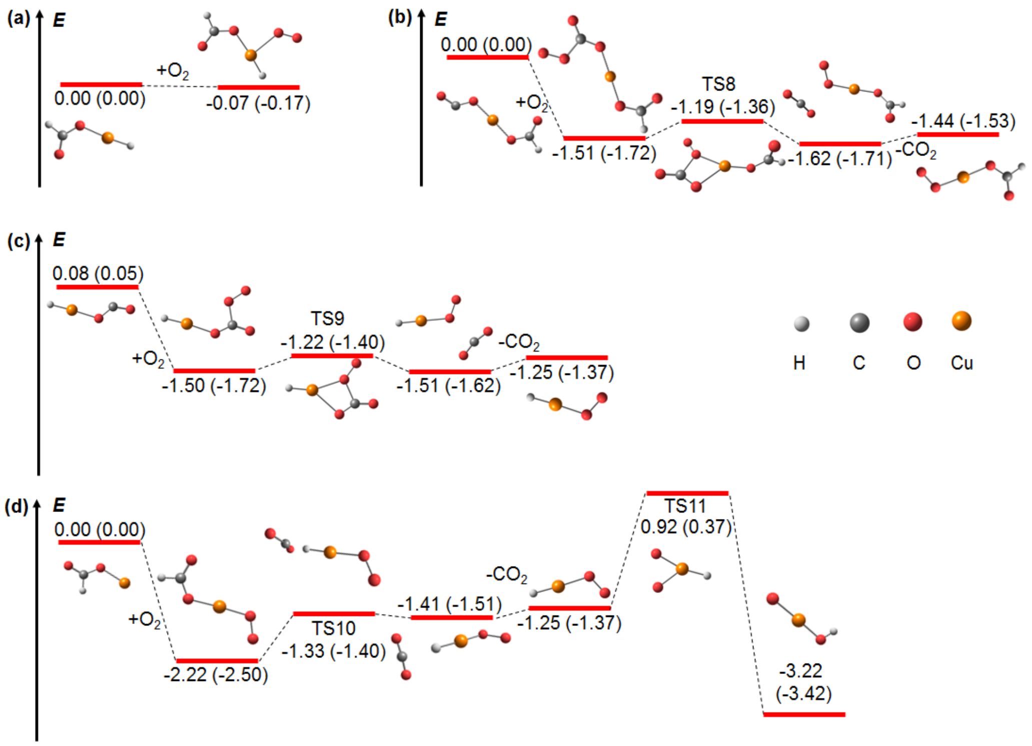

Figure S7. Comparison of the potential energy surface in Figure 5 calculated at the B3LYP/def2TZVP level of theory with zero-point corrected energies in eV with values obtained by BMK/def2TZVP in brackets.

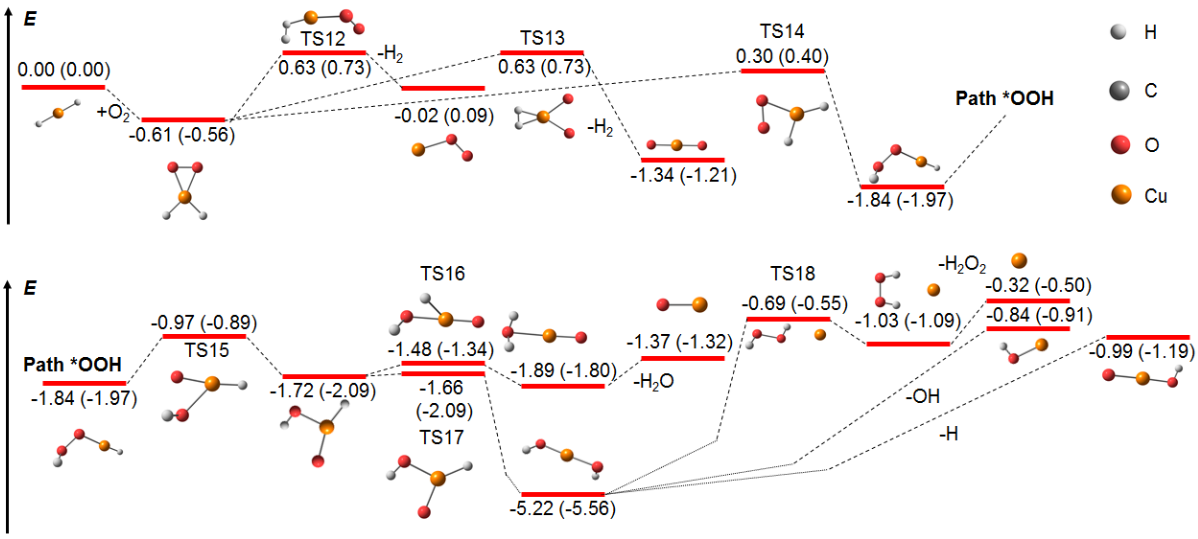

Figure S8. Comparison of the potential energy surface in Figure 6 calculated at the B3LYP/def2TZVP level of theory with zero-point corrected energies in eV with values obtained by BMK/def2TZVP in brackets.

Molecular Orbitals and Charges

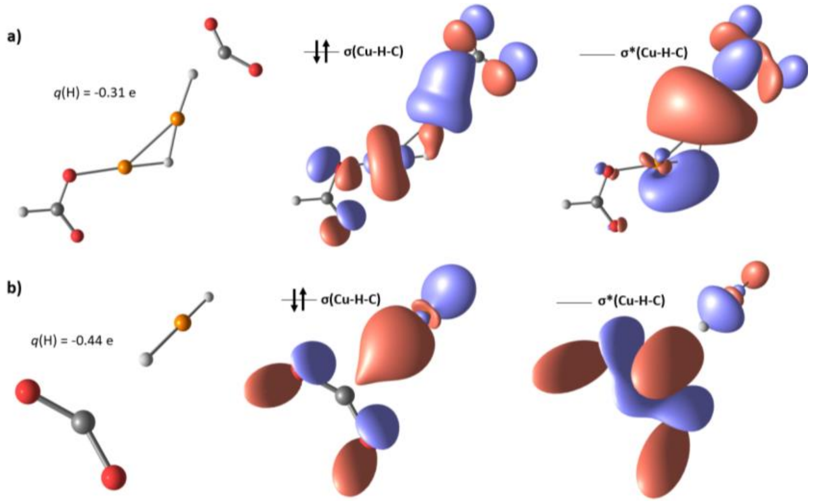

Figure S9. Structure of the hydride transfer transition states along with the characteristic doubly occupied three-center *σ*(Cu-H-C) and *σ**(Cu-H-C) orbitals and the CHELPG charge of the transferring hydrogen atom calculated at the B3LYP/def2ZVP level of Theory. In a) TS6 for reaction $Cu(I)_2(HCO_2)_2H^- \rightarrow Cu(I)_2(HCO_2)H_2^- + CO_2$ and b) the bonding distance *B*(C-H) = 1.8 Å fixated to demonstrate the hydride transfer in the barrierless reaction $Cu(I)(HCO_2)H^- \rightarrow Cu(I)H_2^-$, + CO_2. TS1 and TS4 can be found in [1].

Literature

[1] T. F. Pascher, M. Ončák, C. van der Linde, M. K. Beyer, *ChemPhysChem* **2019**, *20*, 1420.

Cartesian coordinates of optimized ions and molecules (in Å, along with electronic energies (in Hartree) including zero point energy

B3LYP/def2TZVP

CO2
E=-188.658232
O 0.000000 0.000000 1.159737
C 0.000000 0.000000 0.000000
O 0.000000 0.000000 -1.159737

HCu(CO2)-
E=-1829.847146
C 1.630595 -0.287012 0.000000
O 2.830020 -0.097850 0.000000
O 0.643165 0.506757 0.000000
Cu -1.202709 -0.041259 0.000000
H -2.669540 -0.380566 0.000000

Cu(HCO2)-
E=-1829.849931
C -1.591082 0.282800 0.000394
O -2.782053 -0.023493 -0.000383
O -0.589624 -0.511422 0.000302
Cu 1.304049 0.039401 0.000069
H -1.331722 1.370887 0.001196

CuH(HCO2)-
E=-1830.482989
Cu 1.105124 -0.077812 0.000000
O -2.095151 -0.855049 -0.000000
C -1.755818 0.324703 -0.000000
O -0.586345 0.838348 -0.000000
H -2.558776 1.105306 -0.000000
H 2.497053 -0.663358 0.000000

Cu(HCO2)2-
E=-2019.175428
C -2.774974 -0.323672 0.000161
O -3.026758 0.874543 0.000263
O -1.635646 -0.914947 -0.000086
Cu -0.000014 -0.000151 -0.000173
O 1.635446 0.914870 -0.000094
C 2.774920 0.323973 0.000146
O 3.027078 -0.874205 0.000264
H -3.618739 -1.052843 0.000155
H 3.618513 1.053326 0.000242

Cu(HCO2)2
E=-2019.059259
C -2.321774 -0.000422 -0.000045
O -1.683079 1.089093 -0.000040
O -1.682413 -1.089466 0.000010
Cu -0.000016 0.000454 0.000025
O 1.683195 1.089093 0.000045
C 2.321768 -0.000424 -0.000025
O 1.682359 -1.089531 -0.000039
H -3.416889 -0.000720 -0.000081
H 3.416882 -0.000890 -0.000053

Cu(HCO2)3-
E=-2208.394944
Cu -0.000162 -0.132422 -0.000025
O -1.153513 1.145352 0.883003
C -2.218442 1.376439 0.206155
O -2.538355 0.897093 -0.875589
H -2.893533 2.103095 0.709109
O -0.797497 -1.917444 0.757466
C -0.002538 -2.529220 0.000240
O 0.793487 -1.918873 -0.757068
H -0.003517 -3.632321 0.000219
O 1.155923 1.142896 -0.882988
C 2.221255 1.372187 -0.206159
O 2.540349 0.892454 0.875649
H 2.897607 2.097588 -0.709235

Cu(HCO2)CO2-
E=-2018.536985
C 2.683292 -0.189647 -0.000112
O 1.673392 0.589022 -0.000621
Cu -0.064498 -0.110014 -0.000079
O -1.767651 -0.884540 0.000308
C -2.839333 -0.174659 0.000657
O -2.957134 1.042942 0.000812
O 3.871303 0.033258 -0.000311
H -3.755008 -0.809357 0.000829

Cu2H(HCO2)2-, iso2
E=-3660.383812
H -4.816415 1.097942 0.026149
Cu -1.252321 -0.116850 0.005583
C -3.979052 0.364357 0.021099
O -4.234048 -0.833393 0.022342
Cu 1.252326 -0.117130 -0.006516
O 2.829640 0.936695 -0.012588
C 3.979213 0.363248 -0.018883
O 4.233836 -0.834577 -0.021913
O -2.829308 0.937451 0.014475
H 4.816809 1.096580 -0.021709
H -0.000123 -1.065519 -0.000928

Cu2H(HCO2)2-, iso1
E=-3660.367725
O -3.956667 0.278259 -0.001911
H -4.031172 -1.737987 -0.001671
C -3.407529 -0.816193 -0.001370
O -2.151430 -1.088116 -0.000334
Cu -0.890487 0.292057 0.000338
O 0.265230 1.780984 0.001132
C 1.520038 1.856155 -0.000818
H 1.919004 2.886377 -0.000809
O 2.370411 0.943065 -0.002791
Cu 2.235837 -0.992852 0.000505
H 2.304096 -2.490326 0.003146

Cu2(HCO2)3- iso 1
E=-3849.049192
C -0.808627 2.458611 0.000225
O -0.640726 1.944055 1.131976
Cu -0.004141 -0.000145 1.268450
O -1.364817 -1.530283 1.127378
C -1.726833 -1.928543 -0.005440
O -1.366943 -1.521617 -1.135890
Cu 0.004384 -0.000292 -1.267877
O -0.638839 1.946153 -1.131849
O 2.008842 -0.415154 -1.128494
C 2.534756 -0.529488 0.004649
O 2.002505 -0.422216 1.135222
H -1.156525 3.512313 0.001252
H 3.621117 -0.755856 0.006714
H -2.467561 -2.754757 -0.007932

Cu2(HCO2)3- iso 2
E=-3849.053933
O -4.459382 -0.904521 0.012038
C -3.555415 -1.729687 0.003790
H -3.803635 -2.813304 -0.007310
O -2.284645 -1.527742 0.006499
Cu -1.652212 0.227625 0.024106
O -1.132627 2.038716 0.042113
C 0.000019 2.574247 0.047297

H 0.000013 3.676384 0.058265
O 1.132671 2.038732 0.041751
Cu 1.652276 0.227643 0.023965
O 2.284680 -1.527736 0.006538
C 3.555446 -1.729709 0.004208
H 3.803645 -2.813326 -0.007244
O 4.459430 -0.904561 0.012435

Cu2(HCO2)5-
E=-4227.504360
C -0.000230 -0.000078 2.568759
O 1.128422 0.009020 2.027023
Cu 1.499231 0.002173 0.086097
C 3.551192 -0.002165 -1.453690
O 2.295459 -0.003971 -1.698809
O -1.128588 -0.009057 2.026385
Cu -1.499247 -0.002085 0.085489
C -3.551841 0.002292 -1.453020
O -4.060180 -0.002405 -0.331545
O -1.134942 1.969289 -0.021514
C -0.002201 2.503070 -0.068095
O 1.132187 1.971791 -0.034891
O -1.131458 -1.971575 -0.036249
C 0.002904 -2.502968 -0.068325
O 1.135654 -1.969314 -0.020411
O -2.296313 0.004172 -1.699112
O 4.060488 0.002460 -0.332670
H -0.000555 -0.000089 3.671442
H -0.003869 3.604273 -0.148350
H 0.004553 -3.604163 -0.148691
H 4.190324 -0.005115 -2.358606
H -4.191739 0.005270 -2.357384

H2
E=-1.169577
H 4.310818 -1.922111 -1.982526
H 3.707540 -1.636695 -2.311341

H
E=-0.502154
H 0.000000 0.000000 0.000000

TS5
E=-3660.359772
Cu 0.601661 0.001388 -0.260415
Cu -1.602601 -0.990765 0.143329
H -0.429234 -1.933112 0.019667
O 2.456174 -0.263817 -0.799128
C 3.396706 -0.045967 0.037561
H 4.407805 -0.235029 -0.398368
O 3.322329 0.327375 1.205295
O -0.720436 1.649493 -0.172907
C -1.951794 1.631379 0.023903
H -2.467008 2.610587 0.042422
O -2.700551 0.626779 0.206932

TS4
E=-3849.017736
H -1.920825 -1.170680 -0.138563
Cu 1.566234 0.199300 0.009370
Cu -1.425452 0.337530 -0.090718
C -3.277281 -1.338544 0.058633
O -3.632815 -2.128323 -0.779354
O -3.684580 -0.706299 1.012077
O -0.969626 2.197959 -0.116248
C 0.189589 2.674373 -0.101101
O 1.285517 2.067750 -0.055038
O 1.965701 -1.621793 0.096283
C 3.206687 -1.964482 0.078505
O 4.192891 -1.242767 0.011388
H 0.255610 3.773465 -0.132035
H 3.335393 -3.066529 0.133506

TS1
E=-2019.142816 -2019.142816
H 1.298509 0.063356 0.104186
Cu -0.222176 -0.117436 -0.188268
C 2.846337 0.090150 0.138661
O 3.165233 -0.872794 0.764640
O 3.160111 1.069344 -0.464270
O -2.024917 -0.433019 -0.703891
C -3.022825 -0.016599 -0.012627
O -3.025389 0.617552 1.034729
H -3.997801 -0.298760 -0.475220

CuH2-
E=-1641.776028
Cu 0.000000 0.000000 -0.000144
H 0.000000 0.000000 -1.568098
H 0.000000 0.000000 1.572280

Cu2H3-
E=-3282.995008
Cu -1.324344 0.008110 0.000019
H -2.692423 -0.700747 -0.000302
Cu 1.324329 0.008046 0.000006
H 2.692948 -0.699665 -0.000110
H -0.000101 0.931875 -0.000316

Cu2(HCO2)4
E=-4038.166285
C 1.783746 1.783746 0.000000
O 1.394576 1.394576 -1.130017
Cu 0.000000 0.000000 -1.269296
O 1.394576 -1.394576 -1.130017
C 1.783746 -1.783746 0.000000
O 1.394576 -1.394576 1.130017
Cu 0.000000 0.000000 1.269296
O -1.394576 -1.394576 1.130017
C -1.783746 -1.783746 0.000000
O -1.394576 -1.394576 -1.130017
O 1.394576 1.394576 1.130017
O -1.394576 1.394576 1.130017
C -1.783746 1.783746 0.000000
O -1.394576 1.394576 -1.130017
H 2.560479 2.560479 0.000000
H -2.560479 2.560479 0.000000
H -2.560479 -2.560479 0.000000
H 2.560479 -2.560479 0.000000

Cu2(HCO2)H2-
E=-3471.690905
C -3.029384 0.363151 0.000079
O -1.867197 0.906260 0.000158
Cu -0.270906 -0.124215 -0.000372
Cu 2.301375 -0.001945 0.000178
O -3.323161 -0.826356 0.000570
H -3.847466 1.120496 -0.000514
H 1.008116 -1.004057 -0.001048
H 3.654926 0.724053 0.000892

Cu3(HCO2)7-
E=-6246.603899
O -2.856569 1.648539 1.385857
C -3.913686 2.298200 1.070071
O -4.531091 2.182431 0.011530
H -4.259078 3.008534 1.846179
Cu -2.553278 0.524364 -0.181631
O -3.351426 -1.059061 0.730055
C -2.739988 -2.134560 0.931594
H -3.342054 -2.936956 1.392520
H -1.830479 -1.495892 -3.421343
O -1.549769 -2.432199 0.683509
O -0.612004 -1.539929 -1.846595
Cu -0.071477 -1.225535 0.021365
Cu 2.926380 0.488409 0.105796
O 1.651355 -2.497213 -0.297157
C 2.843633 -2.314065 -0.562281
H 3.437961 -3.193513 -0.874015
O 3.514987 -1.237515 -0.532399
H 1.428946 -0.520201 3.556502
O 0.380557 -0.910673 1.915437
C 1.427719 -0.491215 2.453366
O 2.481148 -0.045077 1.937349

O 3.142283 2.535064 0.480014
C 3.688230 2.625384 -0.649442
O 3.836839 1.613522 -1.382738
H 4.038527 3.606431 -1.005615
O -1.165008 1.821175 -0.871937
C 0.068361 1.663890 -0.831741
O 0.710529 0.629471 -0.489166
H 0.681389 2.526755 -1.131304
C -1.670836 -1.145774 -2.388298
O -2.568850 -0.411245 -1.919398

Cu4(HCO2)9-
E=-8265.704038
H -5.668463 -3.195950 1.230629
C -5.212717 -2.225705 0.983478
O -5.277042 -1.790719 -0.195947
O -4.628527 -1.567560 1.883185
Cu -4.199496 -0.133681 0.456292
O -4.606609 1.040939 -1.045494
C -3.878206 1.916708 -1.577488
H -4.393921 2.563871 -2.307779
O -2.668755 2.159431 -1.399824
O -3.740435 1.300744 1.723662
C -2.702590 1.975111 1.903043
O -1.647721 2.017379 1.228135
H -2.717319 2.631569 2.789508
Cu -1.142077 1.198704 -0.506535
O -2.137471 -0.748081 -0.102744
C -1.746721 -1.923376 -0.321863
O -0.585523 -2.305830 -0.564509
H -2.516532 -2.712353 -0.305890
O -0.500089 0.878269 -2.342679
C 0.354844 0.133428 -2.873775
O 1.061814 -0.773377 -2.379557
H 0.505736 0.298600 -3.954345
O 0.719661 0.850774 0.192809
C 1.263803 1.599679 1.064243
O 2.441638 1.525540 1.453785
H 0.634490 2.375221 1.516557
O 1.455258 -2.033972 1.296373
C 2.413760 -1.816611 2.079571
H 2.374492 -2.380853 3.026121
O 3.395258 -1.058123 1.940699
O 4.780852 -0.982536 -0.515283
C 4.216183 -1.948633 -1.073258
O 2.998246 -2.236775 -1.118668
H 4.886166 -2.650141 -1.599445
Cu 1.235932 -1.456413 -0.558501
Cu 3.772431 0.329024 0.570695
O 4.322702 1.784987 -0.607344
C 5.355691 2.292054 -0.040548
H 5.797575 3.141942 -0.597515
O 5.853600 1.915760 1.017333

TS7
E=-3471.655963
H 1.544779 0.004695 0.103204
Cu 0.035371 -0.025585 -0.356685
C 3.207785 0.015700 0.215655
O 3.478806 1.169568 0.218705
O 3.493042 -1.133930 0.259285
Cu -2.517611 0.011501 0.165208
H -1.409545 -0.070607 -1.020127
H -3.713357 0.078105 1.126500

Cu(HCO)H-; *B*(C-H) = 1.8 Å fixated
C 0.000000 0.000000 0.034902
H 0.000000 0.000000 1.834902
Cu 0.000000 0.000000 3.435083
O 0.000000 -1.152657 -0.232889
O 0.000000 1.152657 -0.232889
H 0.000000 0.000000 4.980372

TS6
E=-3660.347230
H -2.564332 0.486835 -0.135862
Cu -1.129292 -0.234099 0.007494
C -3.928342 0.230765 -0.012847
O -4.426456 1.287016 0.280061
Cu 1.380466 -0.121188 0.041806
O 2.875389 1.037123 -0.098077
C 4.050738 0.522359 -0.020434
O 4.354265 -0.653439 0.137404
O -4.171596 -0.926565 -0.249365
H 4.854740 1.285191 -0.114577
H 0.188883 -1.126995 0.165169

TS3b
E=-3849.044421
Cu -0.727498 -0.148558 -0.737036
Cu 1.651388 0.261039 0.594640
O 1.396894 2.126795 0.447625
C 0.544291 2.504357 -0.414337
O -0.223434 1.804334 -1.093897
H 0.500058 3.597083 -0.566305
O 2.038201 -1.585079 0.598807
C 1.388296 -2.277863 -0.250352
O 0.474645 -1.910054 -0.999396
H 1.704916 -3.335811 -0.304502
O -2.592332 -0.798433 -0.483962
C -3.132463 -0.651307 0.660726
O -2.651551 -0.172069 1.687131
H -4.186736 -1.022542 0.694630

TS3a
E=-3849.043813
Cu 0.518561 -0.459719 -0.902562
Cu -0.957356 1.152049 0.572473
O -2.196388 -0.255844 0.890759
C -2.133928 -1.247100 0.102023
O -1.306988 -1.465803 -0.799755
H -2.929802 -2.000080 0.250310
O 0.037090 2.649587 -0.049813
C 0.723317 2.469688 -1.101132
O 0.907749 1.414819 -1.732125
H 1.208219 3.383013 -1.492379
O 2.107207 -1.437129 -0.142516
C 2.279189 -1.170536 1.088875
O 1.614561 -0.423868 1.813178
H 3.153777 -1.697508 1.541509

Cu2(HCO2)3- LM TS3a-TS3b
E=-3849.044416
Cu 0.795155 0.001115 -0.539505
Cu -1.833733 -0.002446 0.253583
O -1.881044 -1.888858 0.185385
C -0.927130 -2.442446 -0.447140
O 0.076380 -1.909906 -0.943532
H -1.030572 -3.536554 -0.559221
O -1.885605 1.883891 0.186455
C -0.933061 2.440174 -0.445747
O 0.071842 1.910404 -0.942312
H -1.039086 3.534107 -0.557072
O 2.737812 0.002395 -0.011907
C 2.974402 0.002841 1.238412
O 2.173609 0.002549 2.174841
H 4.066550 0.003593 1.479515

Cu(OH)2-
E=-1792.353975
O 0.251311 -0.441188 1.332692
Cu 1.842615 0.079639 0.559418
O 3.412803 0.579256 -0.268655
H -0.134337 0.357030 1.710463
H 4.120161 0.364897 0.349711

HCuO4-
E=-1980.291160
C -1.118587 0.667604 -0.000243
O 0.098741 0.930139 -0.001050
Cu 1.725037 -0.126038 0.000355
O -2.131422 1.309200 0.000852
H 3.051431 -0.828951 0.001399
O -1.262619 -0.877641 -0.001415

O -2.500645 -1.301573 0.000797

O2CuH(HCO2)-
E=-1980.868137
C -2.255361 0.132023 -0.306361
O -1.293347 -0.611336 -0.703946
Cu 0.410681 -0.564986 0.278069
O -2.277988 0.926292 0.628566
H -3.176906 0.004508 -0.924205
H 0.580339 -1.729067 1.291330
O 2.171805 0.359516 0.251264
O 1.593254 1.076454 -0.675460

Cu-
E=-1640.582145
Cu 0.000000 0.000000 -0.000144

Cu(HCO2)2H-
E=-2019.712592
C 2.881460 0.219620 0.000188
O 4.009968 -0.243508 0.001040
O 1.782433 -0.461059 -0.000627
Cu -0.000053 0.148128 -0.000377
O -1.782457 -0.461432 -0.000260
C -2.881276 0.219550 0.000463
O -4.009912 -0.243291 0.000644
H 2.734685 1.320514 -0.000027
H -2.734327 1.320424 0.000966
H -0.000165 1.702639 -0.000276

Cu(HCO2)2H-+O2
E=-2170.099385
C 1.657149 -1.423375 0.000291
O 0.835120 -0.426718 0.015531
Cu -1.048942 -0.429599 -0.003591
O -2.538150 0.721701 0.010773
C -3.799100 0.436903 -0.007913
O -4.714651 1.242752 0.003753
O 2.875149 -1.346636 0.015072
H 1.166879 -2.419239 -0.028902
H -4.019651 -0.651248 -0.037080
H -1.548300 -1.900564 -0.046211
O 4.761093 1.358786 -0.607604
O 4.740452 1.368646 0.595233

Cu(HCO2)2-+O2
E=-2169.562182
C -2.519681 1.177752 0.264486
O -1.525731 0.583145 0.820165
Cu 0.120624 0.369130 -0.053951
O 1.760571 0.141389 -0.938086
C 2.880467 0.127854 -0.308980
O 3.092167 0.249774 0.890301
O -2.591317 1.680764 -0.849085
H -3.409975 1.209920 0.933471
H 3.742223 -0.017863 -1.000278
O -0.323233 -2.354211 -0.196367
O -1.213038 -2.886857 0.435193

Cu(HCO2)(CO4)-
E=-2168.978641
C 2.570396 -0.628922 0.011777
O 3.744367 -0.399730 0.009822
O 1.535853 0.077298 0.002575
Cu -0.282880 -0.414476 0.005956
O -2.081330 -0.917828 0.009203
C -3.021270 -0.039547 -0.001134
O -2.932502 1.180084 -0.013900
H -4.028799 -0.513225 0.002716
O 2.127495 -2.102635 0.029288
O 3.117356 -2.959026 0.040124

Cu(HCO2)HO2-
E=-1980.871597
C 2.214185 -0.819004 -0.000189
O 0.936615 -0.876484 -0.000539
Cu -0.184168 0.705966 -0.000192
O 2.945518 0.166805 0.000860
H 2.684881 -1.832290 -0.000966
H -0.952945 2.009286 -0.000087
O -1.945600 -0.783315 0.000429
O -3.146054 -0.474004 0.000220

O2Cu(HCO2)-
E=-1980.317651
O -1.719228 -5.464894 0.000245
C -0.658475 -4.854306 0.001268
H 0.296699 -5.430479 0.000137
O -0.448807 -3.587466 0.000943
Cu -1.817076 -2.303123 0.000509
O -3.112584 -0.957108 0.000225
O -2.598803 0.276703 -0.001403

O2CuH-
E=-1791.623489
O 1.104648 -0.255057 0.000359
O -0.234034 -0.233894 0.000405
Cu -1.048007 -1.973149 -0.000673
H -1.752060 -3.315707 -0.001503

OHCuO-
E=-1791.696243
H 1.137531 0.247238 0.000675
O 0.326253 -0.271864 0.000367
Cu 0.800643 -2.046198 -0.000764
O 1.203701 -3.748007 -0.001847

CuO2-
E=-1790.993036
O 1.119719 -0.152564 0.000423
O -0.196599 -0.232045 0.000405
Cu -0.988511 -2.072512 -0.000737

OCuO-
E=-1791.041869
Cu 1.117423 -0.157391 0.000421
O -0.593943 -0.251511 0.000403
O 2.828412 -0.071241 0.000434

CuO-
E=-1715.770732
O 0.695313 -0.440342 0.000252
Cu -0.638143 -1.675035 -0.000495

CuOH-
E=-1716.433206
O 0.987621 -0.531799 0.000371
Cu -0.642609 -1.497220 -0.000475
H 0.681154 0.384390 0.000350

HCuO-
E=-1716.404054
H -0.109542 0.275796 -0.344451
Cu 0.174217 -0.209222 1.091558
O 0.505764 -0.775925 2.769410

Cu(HCO2)3-+O2
E=-2358.781709
C -1.140246 -1.156807 -1.506911
Cu 0.770125 0.122758 -0.101516
O 0.597103 1.994794 -1.025518
C 0.945896 2.506255 0.068460
O 1.205019 1.801296 1.076140
O -1.933023 -0.677860 -0.703239
O 0.133113 -1.010977 -1.534516
O 1.214231 -1.283516 1.149620
C 2.454223 -1.606231 1.084794
O 3.313042 -1.137488 0.346636
O -4.417288 0.466981 1.019912
O -4.961855 -0.316054 0.286242
H -1.514376 -1.801306 -2.331973
H 1.026705 3.603538 0.145999
H 2.723969 -2.408481 1.805999

H2O2
E=-151.591559
O 0.015516 -0.029632 -0.248600
O -0.040009 -0.013097 1.200693
H 0.973191 -0.021093 -0.389279
H -0.472810 0.839493 1.351656

H2O
E=-76.441817
O -0.019590 0.000000 -0.013852
H 0.016029 0.000000 0.948111
H 0.899231 0.000000 -0.300921

O2
E=-150.386033
O -3.044107 -0.920047 -0.000796
O -2.719010 0.239422 0.000017

OH
E=-75.759756
O -0.879094 0.000000 2.475555
H -0.941747 0.000000 3.450273

TS16
E=-1792.216348
O -0.656334 2.636206 0.729624
Cu -0.010803 1.077363 0.666727
O 0.731343 -0.740226 0.581857
H -0.497246 -0.209092 0.045494
H 0.391061 -1.145869 1.391082

Cu(H2O)O-
E=-1792.231335
O -0.702395 2.753527 0.902389
Cu 0.038111 1.244884 0.673816
O 0.629627 -0.953808 0.506793
H -0.169040 -0.781413 -0.034187
H 0.317719 -0.750808 1.413973

TS17
E=-1792.222970
H 0.036738 0.027302 -0.065492
O 0.092026 -0.041751 0.893721
Cu 1.904953 0.126651 1.342462
O 2.472166 0.323314 -0.364711
H 2.614768 0.093709 2.786670

TS18
E=-1792.187354
O 0.732391 0.071777 0.313164
Cu -0.988548 0.225004 2.328715
O 1.231656 -0.089726 -1.234799
H 1.444551 -1.027115 -1.156454
H -0.228549 0.201343 0.184830

TS9
E=-1980.280669
C 0.210424 0.000131 0.034811
O 0.027744 0.003571 1.212648
O 1.054435 -0.002134 -0.842976
Cu -0.205198 -0.008923 -2.815931
O -1.207700 -0.003236 -0.889172
O -2.364692 -0.002153 -0.287752
H -0.101051 -0.013643 -4.324230

tsco4_scan_2.2.10o1.com.log
E=-1980.291480
C 0.565634 -0.000621 0.543152
O -0.191788 -0.001587 1.431365
O 1.527837 0.000259 -0.122725
Cu -0.655506 -0.004550 -3.111739
O -0.940760 -0.000132 -1.196000
O -2.217051 0.001694 -0.832866
H -0.420405 -0.021451 -4.603789

TS12
E=-1792.139039
O 0.000313 0.241778 0.068587
O 0.197758 -0.345718 1.272098
Cu 1.773285 0.145195 2.190065
H 3.245148 0.197159 2.734761
H 3.439943 -0.470202 2.101525

TS13
E=-1792.139068
H -2.050316 -0.378046 -0.000004
Cu -0.531245 -0.082155 -0.000001
H -1.947415 0.541963 -0.000001
O 1.100928 0.782476 0.000003
O 0.869813 -1.285717 -0.000003

TS14
E=-1792.150896
O -0.008779 -0.000012 -0.049419
O -0.302144 -0.000003 1.380848
Cu 1.636184 -0.000024 0.931388
H 3.020101 -0.000033 1.535637
H 1.392767 -0.000031 -0.591400

CuH(OOH)-
E=-1792.229531
O -0.096664 0.284139 -0.360052
O 0.132765 -0.195728 1.036390
Cu 1.964392 0.003896 1.477178
H 3.419758 0.115287 1.897914
H 0.088169 -0.530716 -0.841635

Cu(H2O2)-
E=-1792.199791
O -0.126984 -0.596598 0.219806
O 0.967156 -1.346250 0.832028
Cu 0.666909 1.267938 2.841704
H 1.186447 -0.764779 1.604430
H -0.199498 0.173541 0.839522

TS8
E=-2168.966849
O -0.075364 -0.526909 0.116248
C 0.009512 -0.013691 1.225125
H 1.016957 0.221517 1.638722
O -0.932613 0.315187 2.031992
Cu -2.736968 -0.000038 1.519741
O -4.562976 -1.052878 1.527584
O -5.176428 -2.145524 1.886781
C -5.449772 0.268648 0.919911
O -6.615455 0.070888 0.805904
O -4.548805 1.078871 0.778714

Cu(HCO2)O2-+CO2
E=-2168.982676
O 0.093082 -0.237440 0.004225
C 0.227034 -0.090859 1.210993
H 1.239351 0.130249 1.622062
O -0.659432 -0.153509 2.139819
Cu -2.469653 -0.502890 1.806828
O -4.312763 -0.795712 1.621227
O -4.738287 -1.929236 2.176601
C -6.114141 0.649046 0.616143
O -6.950319 -0.156308 0.669414
O -5.400785 1.560731 0.493411

TS10
E=-1980.284868
O -0.205727 -0.410882 0.163697
O 0.041729 0.346535 1.235234
Cu 1.802496 0.132797 1.914082
H 3.230125 0.000662 2.516836
C 4.672644 -0.026669 3.263207
O 5.310979 0.796074 2.692773
O 4.553359 -0.862089 4.098428

HCu(O2)-+CO2
E=-1980.287668
O -0.493848 0.126001 0.201830

O -0.007164 0.303172 1.436285
Cu 1.805695 -0.279946 1.636413
H 3.238629 -0.733602 1.850040
C 4.940340 0.166573 3.558726
O 5.371906 0.945853 2.813565
O 4.606050 -0.577624 4.385400

TS15
E=-1792.197885
H -0.836695 0.200804 -1.379274
O 0.253385 -0.051176 1.801420
H 1.101237 0.357696 2.000009
Cu -0.507726 0.817369 -0.004441
O -0.199002 1.777968 1.500441

HCu(OH)O-
E=-1792.225214
H -0.777440 0.069811 -1.015010
O 0.505050 -0.360068 1.281384
H 0.674329 0.145685 2.086364
Cu -0.393915 0.920849 0.220018
O -0.306826 2.406385 1.259399

TS11
E=-1791.543999
Cu 0.071353 -0.001065 0.037579
H -0.032950 0.000358 1.600190
O 1.587635 -0.000021 -1.000814
O -0.284691 -0.003337 -1.748263

CuH2O2-
E= -1792.184652
Cu -0.538376 -0.000055 0.000003
H -1.591766 -1.069095 -0.000028
H -1.591253 1.069949 -0.000019
O 1.174663 0.713483 -0.000003
O 1.174828 -0.713389 -0.000001

BMK/def2TZVP

CO2
E=-188.569891
O 0.000000 0.000000 1.153125
C 0.000000 0.000000 0.000000
O 0.000000 0.000000 -1.153125

Cu(HCO2)-
E=-1828.921167
C -1.586723 0.280363 0.000124
O -2.770438 -0.025053 -0.000165
O -0.590157 -0.505180 0.000158
Cu 1.301218 0.041065 -0.000035
H -1.330222 1.368800 0.000320

HCu(CO2)-
E=-1828.919295
C 1.227930 1.094960 0.000000
O 1.830165 2.140664 0.000000
O 0.000000 0.825120 0.000000
Cu -0.712387 -0.963704 0.000000
H -1.349676 -2.348607 0.000000

CuH(HCO2)-
E=-1829.552602
Cu -1.094481 -0.074870 -0.000004
O 2.053936 -0.864753 -0.000008
C 1.746059 0.317114 0.000014
O 0.595256 0.849323 0.000011
H 2.567888 1.074915 0.000003
H -2.497820 -0.682919 -0.000012

Cu(HCO2)H- O2
E=-1979.869428
C 2.155508 0.222121 0.220191
O 1.287367 -0.501628 0.796189
Cu -0.447105 -0.524719 -0.140398
O 2.020663 0.889453 -0.794952
H 3.144335 0.219228 0.733468
H -0.515478 -1.851390 -0.965952
O -2.203363 0.450611 -0.230433
O -1.429150 1.101098 0.602055

CuH2-
E=-1640.931903
Cu 0.000000 -0.000016 -0.000000
H 1.575593 -0.216228 -0.000000
H -1.575593 0.216695 0.000000

CuH2O2-
E=-1791.253329
Cu 0.000000 0.636884 0.000000
H -1.299664 1.558906 0.000000
H 1.300272 1.558250 0.000000
O 0.656538 -1.348773 0.000000
O -0.656614 -1.349575 0.000000

Cu(HCO2)2H- O2
E=-2169.005399
C -2.866272 -0.706786 0.095535
O -1.760947 -0.263248 -0.384968
Cu -0.000404 -0.659997 0.124048
O 1.760337 -0.263995 -0.384896
C 2.865439 -0.708195 0.095507
O 3.978398 -0.411365 -0.283267
O -3.979082 -0.409472 -0.283297
H -2.742275 -1.425068 0.933397
H 2.741082 -1.426591 0.933218
H -0.000627 -1.638778 1.342783
O 0.001083 2.314893 0.644972
O 0.002526 3.048217 -0.302675

Cu(HCO2)2-
E=-2018.162858
C -2.761408 -0.319458 -0.000020
O -2.994104 0.876598 0.000008
O -1.636179 -0.915377 -0.000019
Cu -0.000021 0.000427 -0.000001
O 1.636644 0.915315 0.000014
C 2.761541 0.318771 0.000012
O 2.993547 -0.877420 0.000006
H -3.616888 -1.033022 -0.000016
H 3.617427 1.031848 0.000018

Cu(HCO2)2
E=-2018.065295
C 2.294781 -0.000132 0.000023
O 1.661402 1.083445 -0.000099
O 1.661159 -1.083557 -0.000081
Cu 0.000000 0.000137 0.000014
O -1.661416 1.083443 0.000042
C -2.294780 -0.000140 -0.000016
O -1.661146 -1.083559 0.000059
H 3.390661 -0.000248 0.000180
H -3.390660 -0.000271 0.000014

Cu(HCO2)2- O2
E=-2168.473353
C 1.140040 1.439438 -0.084683
O -0.120796 1.595656 -0.001631
Cu -1.327611 0.160789 -0.017031
O -2.534782 -1.273758 -0.032187
C -3.795527 -1.114794 0.050251
O -4.431551 -0.080175 0.148276
O 1.778383 0.406187 -0.183670
H 1.688164 2.408855 -0.058942
H -4.345608 -2.083120 0.024916
O 6.284183 -0.407353 0.562713
O 6.160948 -1.107617 -0.401689

Cu(HCO2)3-
E=-2207.306013
Cu -0.000001 0.081576 -0.000006
O -1.157898 -1.197436 -0.898180
C -2.192677 -1.301771 -0.169664
O -2.399632 -0.709754 0.881346
H -2.954971 -2.006190 -0.561385
O -0.710725 1.845139 -0.827941
C 0.000347 2.452668 -0.000012
O 0.711139 1.844910 0.827991
H 0.000440 3.555350 0.000060
O 1.157622 -1.197705 0.898162
C 2.192392 -1.302193 0.169657
O 2.399445 -0.710199 -0.881349
H 2.954575 -2.006734 0.561375

Cu(HCO2)CO2-
E=-2017.526788
C 2.679062 -0.186798 -0.007995
O 1.671249 0.576185 0.009825
Cu -0.068126 -0.115986 0.003660
O -1.774500 -0.882152 -0.000062
C -2.830022 -0.166367 -0.004119
O -2.925884 1.047119 -0.005836
O 3.858971 0.041914 -0.007333
H -3.757265 -0.781956 -0.006211

CuO2HCO2-
E=-1979.323399
O -2.625758 0.972581 -0.000046
C -2.452171 -0.232955 0.000040
H -3.341522 -0.904006 -0.000139
O -1.357338 -0.884136 0.000002
Cu 0.327424 -0.060359 0.000017
O 2.056521 0.673902 0.000036
O 2.996481 -0.255830 -0.000065

CuO2H-
E=-1790.712030
O 0.490290 -1.929280 0.000000
O -0.536266 -1.093846 0.000000
Cu -0.000000 0.756568 -0.000000

H 0.367810 2.244536 -0.000000

OCuOH-
E=-1790.787342
H -0.467466 -2.218709 -0.000000
O 0.362833 -1.739909 -0.000000
Cu 0.000000 0.059964 0.000000
O -0.304400 1.799877 0.000000

CuO2-
E=-1790.080998
O 0.509072 -1.921654 -0.000000
O -0.509072 -1.092043 0.000000
Cu 0.000000 0.831365 -0.000000

OCuO-
E=-1790.128710
Cu -0.000000 0.001622 0.000000
O 1.750159 0.001107 0.000000
O -1.750159 -0.006987 -0.000000

CuO-
E=-1714.893269
O -0.000000 -0.000000 -1.451514
Cu 0.000000 0.000000 0.400418

CuOH-
E=-1715.557517
O 0.024566 1.430241 -0.000000
Cu 0.024566 -0.451792 0.000000
H -0.908955 1.660041 -0.000000

Cu2H(HCO2)2-, Iso.2
E=-3658.540521
H 4.797802 1.090870 0.002872
Cu 1.254581 -0.132494 -0.000482
C 3.947071 0.374574 0.001762
O 4.179995 -0.821979 0.000869
Cu -1.254589 -0.132471 -0.001439
O -2.812348 0.954463 -0.001411
C -3.947069 0.374553 0.001185
O -4.179939 -0.822010 0.003539
O 2.812325 0.954444 0.001637
H -4.797832 1.090812 0.001183
H -0.000008 -1.111781 -0.003108

Cu2H3-
E=-3281.317706
Cu 1.308138 0.008924 0.000105
H 2.668031 -0.755824 0.000052
Cu -1.308140 0.008933 -0.000109
H -2.667941 -0.755988 -0.000028
H -0.000017 0.993956 0.000088

Cu2H(HCO2)2-, Iso.1
E=-3658.523790
O -3.873536 0.341606 -0.000071
H -4.020700 -1.663054 -0.000027
C -3.364076 -0.765640 -0.000028
O -2.126152 -1.069461 0.000010
Cu -0.843135 0.290192 0.000005
O 0.314890 1.782948 -0.000007
C 1.561616 1.841261 -0.000035
H 1.981307 2.862940 -0.000058
O 2.385812 0.913944 -0.000040
Cu 2.124469 -1.010181 0.000037
H 2.087363 -2.526224 0.000122

Cu2(HCO2)3- Iso.1
E=-3847.128704
C -0.800831 2.448046 -0.002768
O -0.634448 1.936576 1.121093
Cu -0.003405 0.001593 1.283072
O -1.360002 -1.518208 1.121015
C -1.720842 -1.916966 -0.002779
O -1.361240 -1.513641 -1.125338
Cu 0.004298 -0.001597 -1.282762
O -0.632321 1.934520 -1.125133
O 1.996117 -0.418173 -1.119344
C 2.520530 -0.531111 0.005183
O 1.989918 -0.421193 1.126881
H -1.147612 3.502023 -0.003871
H 3.606585 -0.758238 0.007704
H -2.462189 -2.742517 -0.004042

Cu2(HCO2)3- Iso.2
E=-3847.127996
Cu -1.621967 0.228853 0.007907
Cu 1.621971 0.228980 0.010913
O -2.240752 -1.530725 0.025290
C -3.501198 -1.728088 -0.006043
H -3.762447 -2.807718 0.007484
O -4.392799 -0.898894 -0.046862
O -1.122829 2.047153 -0.005325
C -0.000117 2.584318 -0.007303
H -0.000135 3.686689 -0.015249
O 1.122613 2.047220 -0.001512
O 2.240982 -1.530504 0.028459
C 3.501283 -1.728014 -0.007355
O 4.392806 -0.898971 -0.052599
H 3.762483 -2.807653 0.006598

Cu2(HCO2)4
E-4036.185188
C 1.775182 1.775182 0.000000
O 1.383421 1.383421 -1.120057
Cu 0.000000 0.000000 -1.281679
O 1.383421 -1.383421 -1.120057
C 1.775182 -1.775182 0.000000
O 1.383421 -1.383421 1.120057
Cu 0.000000 0.000000 1.281679
O -1.383421 -1.383421 1.120057
C -1.775182 -1.775182 0.000000
O -1.383421 -1.383421 -1.120057
O 1.383421 1.383421 1.120057
O -1.383421 1.383421 1.120057
C -1.775182 1.775182 0.000000
O -1.383421 1.383421 -1.120057
H 2.551742 2.551742 0.000000
H -2.551742 2.551742 0.000000
H -2.551742 -2.551742 0.000000
H 2.551742 -2.551742 0.000000

Cu2(HCO2)5-
E=-4225.431002
C 0.000001 0.000001 2.522655
O -1.101443 0.201466 1.979438
Cu -1.522124 0.042291 0.058499
C -3.579145 -0.059352 -1.319664
O -2.382063 -0.104815 -1.722593
O 1.101443 -0.201464 1.979437
Cu 1.522124 -0.042291 0.058497
C 3.579148 0.059350 -1.319660
O 3.904499 -0.045814 -0.135895
O 1.084701 -1.961702 -0.237456
C -0.040759 -2.478497 -0.108762
O -1.135780 -1.919032 0.088305
O 1.135777 1.919032 0.088299
C 0.040756 2.478497 -0.108763
O -1.084705 1.961703 -0.237453
O 2.382068 0.104814 -1.722593
O -3.904499 0.045813 -0.135900
H 0.000002 0.000001 3.625098
H -0.072360 -3.579120 -0.178749
H 0.072357 3.579120 -0.178751
H -4.357905 -0.119819 -2.102022
H 4.357911 0.119816 -2.102016

Cu2(HCO2)H2-
E=-3469.930437
C 3.005025 0.363863 -0.000010
O 1.860203 0.920275 0.000015

Cu 0.274962 -0.130561 0.000172
Cu -2.284788 0.000378 -0.000187
O 3.270703 -0.826195 0.000095
H 3.840023 1.100454 -0.000149
H -1.006334 -1.047307 0.000352
H -3.626132 0.786345 -0.000595

Cu3(HCO2)7-
E=-6243.548752
O -2.738778 1.820890 1.353359
C -3.789261 2.381736 0.928089
O -4.351701 2.074626 -0.124620
H -4.201108 3.189114 1.559699
Cu -2.536071 0.546973 -0.147244
O -3.203345 -0.975289 0.938171
C -2.668366 -2.097236 1.011956
H -3.272399 -2.885337 1.491936
H -1.958037 -1.517418 -3.361321
O -1.541927 -2.447335 0.612433
O -0.637372 -1.445848 -1.873727
Cu -0.069252 -1.246119 0.011701
Cu 2.875703 0.491503 0.146100
O 1.667513 -2.455184 -0.363896
C 2.851835 -2.260503 -0.635574
H 3.443461 -3.129160 -0.977616
O 3.502235 -1.184331 -0.578562
H 1.471697 -0.718483 3.560200
O 0.391735 -1.000149 1.919375
C 1.444620 -0.625098 2.461624
O 2.479537 -0.160431 1.942424
O 3.048040 2.503272 0.629900
C 3.515739 2.677138 -0.515714
O 3.656516 1.721075 -1.310277
H 3.804860 3.690620 -0.834570
O -1.109642 1.713299 -0.985059
C 0.114310 1.581033 -0.894368
O 0.736943 0.589763 -0.432131
H 0.739204 2.412023 -1.255551
C -1.753757 -1.155904 -2.340546
O -2.669681 -0.507695 -1.800107

H2
E=-1.158059
H -0.000000 -0.000000 0.370754
H 0.000000 -0.000000 -0.370754

H2O
E=-76.397658
O 0.000000 -0.000000 0.115889
H 0.000000 -0.763785 -0.463555
H 0.000000 0.763785 -0.463555

O2
E=-150.310446
O 0.000000 0.000000 0.599243
O -0.000000 0.000000 -0.599243

OH
E=-75.718134
O 0.000000 -0.000000 0.108017
H -0.000000 0.000000 -0.864133

TS1
E=-2018.122803
c -0.550491 -2.799368 0.000000
h -0.683347 -1.161005 0.000000
cu 0.088826 0.197500 -0.000000
o 1.154169 1.776333 -0.000000
c 0.635682 2.938560 -0.000000
o -0.543301 3.248302 -0.000000
h 1.402098 3.747572 -0.000000

TS7
E=-3469.888284
H 1.525328 0.006591 0.339318
Cu 0.051689 -0.022946 -0.257209
C 3.193990 0.014168 0.168769
O 3.457874 1.161092 0.128683
O 3.473994 -1.129651 0.173759
Cu -2.502129 0.010790 0.126042
H -1.354511 -0.064612 -1.048960
H -3.726965 0.074016 1.081343

TS2
E=-1829.508244
cu 0.000000 0.000000 1.559067
h 0.000000 0.000000 3.132393
xx 0.000000 0.000000 -2.066461
c 0.000000 1.150637 -2.244061

TS6
E=-3658.497958
C -4.104988 0.227078 0.142597
O -4.440080 -0.631550 0.883570
O -4.368596 1.080638 -0.632109
Cu -1.092066 -0.252790 -0.152995
Cu 1.434151 -0.128172 -0.090474
O 2.893719 0.852499 0.627461
C 4.066532 0.458365 0.320915
O 4.379839 -0.483782 -0.386477
H -2.527891 0.288571 0.275735
H 4.865566 1.081288 0.778400
H 0.253537 -0.977040 -0.714164

TS5
E=-3658.517728
Cu 0.627292 0.034838 -0.271120
Cu -1.612696 -0.995035 0.147570
H -0.453565 -1.983961 0.032498
O 2.473630 -0.338061 -0.790398
C 3.372148 -0.082092 0.066235
H 4.400067 -0.320788 -0.294915
O 3.242425 0.376143 1.192321
O -0.715642 1.621517 -0.184805
C -1.939945 1.619763 0.012926
H -2.441255 2.605542 0.026025
O -2.690632 0.630263 0.200927

Cu2H(HCO2)2CO2-
E=-3847.097388
H 2.068807 -0.884249 -0.002832
Cu -1.862554 0.078602 0.001443
Cu 1.344455 0.450299 -0.005171
C 4.679349 -1.188241 0.005708
O 4.733258 -1.149905 1.158003
O 4.721900 -1.245861 -1.146251
O 0.610359 2.242376 -0.008984
C -0.569110 2.629788 -0.007951
O -1.616114 1.951628 -0.004206
O -2.267597 -1.745440 0.007637
C -3.488317 -2.113668 0.007264
O -4.489160 -1.418397 0.003926
H -0.719226 3.723646 -0.010719
H -3.597412 -3.220016 0.010516

TS4
E=-3847.087285
H -1.992662 -1.073685 -0.001408
C -3.497321 -1.301354 0.000067
O -3.818470 -1.384745 1.141844
O -3.822296 -1.376936 -1.141185
Cu -1.398009 0.376702 0.000004
Cu 1.612895 0.169812 -0.000942
O 1.356379 2.044057 0.000337
C 0.282876 2.675492 0.001404
O -0.878252 2.226325 0.001656
H 0.374816 3.773800 0.002231
O 1.980486 -1.658749 -0.002528
C 3.207171 -2.013209 -0.000377
O 4.194456 -1.299707 0.002527

H 3.331378 -3.116544 -0.001415

TS3
E=-3847.121103
cu 0.346868 -1.131991 -0.636323
cu -0.838115 1.001103 0.618010
o -2.315161 -0.217710 0.757312
c -2.389634 -1.191231 -0.021263
o -1.513739 -1.640268 -0.783472
h -3.358567 -1.719440 -0.034342
o 0.414152 2.367725 0.208438
c 0.849686 2.254280 -0.981480
o 0.531279 1.418236 -1.816419
h 1.599079 3.026661 -1.252438
o 2.205942 -1.046742 -0.214401
c 2.414151 -0.809116 1.017446
o 1.585909 -0.669293 1.905743
h 3.493357 -0.730642 1.272034

Cu(HCO2)3- O2
E=-2357.616497
C 0.087294 2.332057 -1.037737
Cu 0.762650 0.097626 0.109124
O 1.090202 0.846691 2.014218
C 0.590106 -0.209777 2.454634
O 0.178370 -1.110083 1.693019
O -0.994470 2.008763 -0.565367
O 1.173514 1.681452 -0.941259
O 0.528606 -1.147181 -1.366874
C 1.633796 -1.762442 -1.478799
O 2.629099 -1.592101 -0.787121
O -4.728574 -0.565864 0.411675
O -4.666842 -0.796539 -0.762434
H 0.173520 3.276604 -1.613188
H 0.509247 -0.351237 3.545166
H 1.653970 -2.516643 -2.292021

Cu4(HCO2)9-
E=-8261.667329
H -5.503453 -3.172934 1.325766
C -5.094551 -2.192293 1.039609
O -5.153282 -1.824919 -0.155026
O -4.573215 -1.464580 1.912466
Cu -4.179994 -0.101436 0.426036
O -4.593230 0.995223 -1.124853
C -3.875592 1.881157 -1.635508
H -4.377578 2.522715 -2.378807
O -2.681899 2.134152 -1.416296
O -3.755116 1.410542 1.599858
C -2.724727 2.076477 1.803033
O -1.644522 2.055723 1.182902
H -2.773855 2.780630 2.649963
Cu -1.151299 1.196530 -0.538392
O -2.141099 -0.701149 -0.079723
C -1.745290 -1.879080 -0.244431
O -0.588787 -2.248302 -0.475396
H -2.514198 -2.667198 -0.183487
O -0.528360 0.830450 -2.365043
C 0.326158 0.091864 -2.887749
O 1.044022 -0.779556 -2.365866
H 0.463509 0.224797 -3.973655
O 0.692645 0.800949 0.162924
C 1.226712 1.453753 1.107133
O 2.393904 1.340382 1.488715
H 0.588479 2.177940 1.627654
O 1.492966 -1.898051 1.348915
C 2.528514 -1.819723 2.042253
H 2.521265 -2.411770 2.971796
O 3.559244 -1.163248 1.821909
O 4.719041 -0.901526 -0.731566
C 4.149947 -1.910265 -1.183256
O 2.951214 -2.231341 -1.081203
H 4.787801 -2.613718 -1.743733
Cu 1.231897 -1.396168 -0.525348
Cu 3.811899 0.313836 0.528156
O 4.262590 1.907241 -0.564230
C 5.264655 2.282000 0.112498
H 5.780923 3.189912 -0.248445
O 5.672580 1.700031 1.117557

Cu(HCO2)2H-
E=-2018.695670
C 2.864439 0.193672 0.000101
O 3.978278 -0.285678 0.000143
O 1.759958 -0.461290 -0.000334
Cu 0.000000 0.182329 -0.000189
O -1.759958 -0.461289 0.000030
C -2.864440 0.193673 0.000298
O -3.978278 -0.285678 0.000499
H 2.739173 1.297235 0.000367
H -2.739175 1.297236 0.000332
H 0.000003 1.745399 -0.000307

Cu(OH)2-
E=-1791.446768
O 0.264064 -0.439164 1.350996
Cu 1.844718 0.088997 0.567949
O 3.412321 0.593832 -0.255579
H -0.160140 0.350292 1.692208
H 4.131590 0.345678 0.328056

HCu(CO4)-
E=-1979.294771
C -1.118889 0.651937 -0.000275
O 0.087033 0.938665 -0.000383
Cu 1.708533 -0.120025 0.000319
O -2.135716 1.277468 0.000020
H 3.045683 -0.844621 0.000837
O -1.233349 -0.859830 -0.000949
O -2.453936 -1.303289 0.000531

CuO2HH-
E=-1791.263030
Cu -0.528030 0.000083 -0.000001
H -1.584647 -1.068099 -0.000025
H -1.584091 1.068824 -0.000020
O 1.162401 0.705847 0.000001
O 1.162463 -0.705761 -0.000003

(HCO2)Cu(CO4)-
E=-2167.900401
C -2.140170 -0.647723 -0.000011
O -3.197552 -1.195569 -0.000193
O -0.956841 -1.037226 0.000191
Cu 0.641645 -0.042061 -0.000153
O 2.215140 0.963609 -0.000583
C 3.357503 0.394933 -0.000028
O 3.611751 -0.795288 0.000797
H 4.193950 1.128126 -0.000379
O -2.127268 0.859462 -0.000294
O -3.307493 1.402385 0.000823

HCu(O2)-+CO2
E=-1979.287154
O -0.548954 -0.042497 0.428500
O 0.150069 0.519554 1.401681
Cu 1.907364 -0.243600 1.559202
H 3.317950 -0.825357 1.719291
C 4.850348 0.168858 3.563853
O 5.307860 0.949434 2.846400
O 4.476969 -0.575964 4.363331

HCuO2-+CO2
E=-1979.291054
C 0.588326 0.000691 0.595736
O -0.212045 0.004059 1.431538
O 1.540975 -0.002341 -0.066378
Cu -0.680428 -0.009897 -3.138434
O -0.927052 -0.003272 -1.219749
O -2.189643 -0.000455 -0.845509
H -0.452171 -0.015171 -4.649807

Cu-

E=-1639.751142
Cu 0.000000 0.000000 -0.000144

H2O2
E=-151.509743
O 0.016277 -0.032872 -0.235881
O -0.042703 -0.013442 1.187925
H 0.968301 -0.015636 -0.385267
H -0.465985 0.837622 1.347693

H
E=-0.498614
H 0.000000 0.000000 0.000000

TS16
E=-1791.291755
O -0.641497 2.649377 0.742643
Cu -0.022550 1.078804 0.661098
O 0.721549 -0.718375 0.587724
H -0.492710 -0.250713 0.034538
H 0.393229 -1.140711 1.388781

OCu(H2O)-
E=-1791.308422
O -0.689668 2.755908 0.899859
Cu 0.029506 1.237413 0.675473
O 0.633456 -0.904460 0.504990
H -0.172196 -0.805757 -0.032846
H 0.312924 -0.770721 1.415308

TS17
E=-1791.319210
H 0.020202 0.024007 -0.048123
O 0.097020 -0.042022 0.905364
Cu 1.899765 0.126950 1.331493
O 2.505787 0.330185 -0.404203
H 2.597876 0.090104 2.808119

TS18
E=-1791.262717
O 0.562192 0.034121 0.329452
Cu -0.808001 0.155707 2.319198
O 1.277219 -0.100107 -1.170912
H 1.469611 -1.035124 -1.066671
H -0.309521 0.326687 0.024389

TS9
E=-1979.282994
C 0.199420 0.000098 0.029424
O -0.008177 0.003804 1.198858
O 1.063899 -0.002263 -0.818146
Cu -0.262830 -0.008986 -2.810953
O -1.168258 -0.003485 -0.887218
O -2.316721 -0.002095 -0.290539
H -0.093371 -0.013460 -4.334028

TS12
E=-1791.215631
O 0.026818 0.233895 0.074689
O 0.155073 -0.346368 1.259305
Cu 1.742308 0.168996 2.218114
H 3.238706 0.195904 2.731201
H 3.493541 -0.484215 2.083728

TS13
E=-1791.215495
o 0.040258 0.000000 -0.000933
cu -0.003828 0.000000 1.852567
o 1.705549 0.000000 1.134629
h -0.472593 0.000000 3.336494
h -1.214068 0.000000 2.830880

TS14
E=-1791.227665
O -1.160307 0.682783 -0.000001
O -1.253928 -0.753014 0.000000
Cu 0.582867 -0.006556 0.000000
H 2.055068 -0.412243 0.000002
H 0.119316 1.452340 -0.000001

CuH(OOH)-
E=-1791.314605
O -0.080653 0.279897 -0.348087
O 0.119912 -0.196476 1.020672
Cu 1.957946 0.002633 1.479253
H 3.432145 0.119775 1.898333
H 0.079070 -0.528951 -0.840377

Cu(H2O2)-
E=-1791.282547
O -0.084841 -0.580933 0.200972
O 0.924537 -1.362367 0.848038
Cu 0.670383 1.329215 2.901076
H 1.304254 -0.718224 1.483342
H -0.320302 0.066161 0.900062

TS8
E=-2167.887155
O -0.121517 -0.729680 0.246472
C -0.010939 -0.028377 1.237572
H 1.005595 0.275414 1.573344
O -0.933248 0.432907 1.984186
Cu -2.728795 -0.009410 1.533862
O -4.534866 -1.011520 1.475275
O -5.118217 -2.135002 1.742149
C -5.442256 0.267892 0.960432
O -6.595415 0.026738 0.843287
O -4.592255 1.127110 0.854143

Cu(HCO2)(O2)-+CO2
E=-2167.899940
O 0.097009 -0.248800 0.024834
C 0.237169 -0.103254 1.225066
H 1.253963 0.104860 1.628449
O -0.645077 -0.154127 2.144416
Cu -2.453334 -0.484855 1.791613
O -4.306230 -0.764880 1.581821
O -4.730580 -1.880937 2.142011
C -6.141869 0.637774 0.627157
O -6.945323 -0.187964 0.705113
O -5.451641 1.556254 0.490242

TS10
E=-1979.282974
c -0.015622 0.051190 -0.002845
o -0.024943 0.097086 1.175298
o 0.531026 0.108646 -1.045954
cu -3.050081 0.016762 -0.770833
o -4.849005 0.452524 -1.231351
o -5.578457 -0.636701 -1.404742
h -1.568664 -0.313085 -0.392549

TS15
E=-1791.275062
H -0.833612 0.196637 -1.388398
O 0.243100 -0.021658 1.805415
H 1.109399 0.344516 1.986747
Cu -0.504245 0.816943 0.006877
O -0.203444 1.766223 1.507514

HCu(OH)O-
E=-1791.318988
H -0.835019 0.123417 -1.134465
O 0.497965 -0.341502 1.355087
H 0.726059 0.171392 2.133858
Cu -0.383657 0.851017 0.217396
O -0.304150 2.378338 1.260280

TS11
E=-1790.648110
Cu 0.057869 -0.001111 0.025984
H -0.020154 0.000468 1.605584
O 1.612415 0.000188 -0.976562
O -0.308783 -0.003609 -1.766314

DF-PBE/6-31G*

Cu2(HCO2)5-
(DF-PBE/6-31G*)
E=-4225.021168
C -0.000120 0.000290 2.567333
O -1.117607 -0.251657 2.020328
Cu -1.352207 -0.054430 0.096308
C -3.234129 0.054779 -1.526042
O -1.963618 0.139404 -1.749121
O 1.117373 0.252079 2.020280
Cu 1.352208 0.054503 0.096348
C 3.234053 -0.055210 -1.526042
O 3.764102 0.098520 -0.404615
O 1.204131 -1.893843 0.238807
C 0.096789 -2.465762 -0.006594
O -1.056046 -1.964326 -0.182386
O 1.056260 1.964378 -0.182667
C -0.096624 2.465802 -0.007189
O -1.203954 1.893889 0.238301
O 1.963527 -0.139741 -1.749079
O -3.764133 -0.098849 -0.404579
H -0.000116 0.000368 3.679511
H 0.143535 -3.575587 -0.072837
H -0.143436 3.575601 -0.073781
H -3.868962 0.131638 -2.445441
H 3.868859 -0.132360 -2.445434

Cu3(HCO2)7-
(DF-PBE/6-31G*)
E= -6243.022766
O -2.391298 1.602621 1.385369
C -3.468503 2.266533 1.112699
O -4.158966 2.128437 0.081352
H -3.759938 3.010955 1.895843
Cu -2.339086 0.496495 -0.223176
O -3.235809 -0.942996 0.718567
C -2.658523 -2.061953 0.887091
H -3.285014 -2.839402 1.377852
H -1.884566 -1.545338 -3.445889
O -1.485380 -2.431248 0.577152
O -0.592801 -1.592312 -1.904907
Cu -0.131387 -1.142387 -0.067225
Cu 2.717766 0.434176 0.246382
O 1.570639 -2.438538 -0.132656
C 2.727571 -2.353089 0.343539
H 3.290936 -3.302206 0.515592
O 3.390928 -1.302995 0.674717
H 0.526218 0.515676 3.401225
O 0.014556 -0.568968 1.787845
C 0.814877 0.226292 2.366798
O 1.902169 0.741519 1.949601
O 3.136276 2.460967 -0.246898
C 3.904413 1.956679 -1.124680
O 4.034626 0.691015 -1.246942
H 4.484728 2.621295 -1.802075
O -1.121820 1.809055 -1.026789
C 0.123701 1.615453 -1.026224
O 0.754934 0.543318 -0.708471
H 0.765964 2.464222 -1.332765
C -1.676543 -1.176463 -2.418229
O -2.557026 -0.403143 -1.930342

Cu4(HCO2)9-
(DF-PBE/6-31G*)
E=-8261.027065
H -6.107160 -2.833400 -0.494880
C -5.463674 -1.950506 -0.293095
O -5.341697 -1.045077 -1.183000
O -4.861298 -1.849152 0.826291
Cu -4.092886 -0.075560 0.088063
O -4.334390 1.594741 -0.814846
C -3.488165 2.542906 -0.915734
H -3.899940 3.486218 -1.340212
O -2.262429 2.565713 -0.606374
O -3.775433 0.730424 1.817999
C -2.690313 1.175922 2.299825
O -1.553524 1.323731 1.749315
H -2.737193 1.487932 3.366456
Cu -1.116808 1.014875 -0.111215
O -2.115726 -0.835691 -0.258630
C -1.655732 -1.999878 0.031875
O -0.450650 -2.331353 0.158676
H -2.422086 -2.790593 0.194494
O -0.544371 1.021227 -1.943678
C 0.190012 0.147661 -2.506176
O 0.818387 -0.842499 -2.024131
H 0.290710 0.275343 -3.606996
O 0.799209 0.660654 0.441842
C 1.393236 1.392639 1.314851
O 2.625865 1.360317 1.577400
H 0.763912 2.098182 1.890030
O 1.857176 -1.946372 1.530945
C 3.010737 -1.769260 2.034569
H 3.215667 -2.358346 2.954349
O 3.958238 -1.022560 1.643536
O 4.546263 -0.775324 -1.034518
C 3.977370 -1.862908 -1.362968
O 2.828405 -2.297809 -1.054750
H 4.583221 -2.528132 -2.016956
Cu 1.302420 -1.344708 -0.220900
Cu 3.671719 0.330417 0.283220
O 3.510213 1.787849 -0.996188
C 4.608046 2.418157 -0.714473
H 4.779227 3.332461 -1.335725
O 5.430212 2.080709 0.160301

Cu6(HCO2)13-
(DF-PBE/6-31G*)
E=-12297.029911
C 6.440219 3.282938 -1.368741
Cu 6.060606 1.379745 -0.042294
O 4.191930 0.620101 -0.746816
C 3.679154 0.850314 -1.893276
O 2.539273 0.473694 -2.294644
Cu 1.271499 -0.345679 -1.024727
O -0.657519 -0.948353 -0.839304
C -1.269386 -1.585132 -1.783800
O -2.498566 -1.821092 -1.845748
Cu -3.751362 -0.847720 -0.625261
O -5.520667 -1.296438 -1.713321
C -6.676421 -0.810762 -1.760758
O -7.160927 0.204027 -1.139808
O 6.720438 2.122938 -1.827192
O 5.962311 3.416287 -0.196170
O 7.251821 -0.137239 -0.026206
C 6.955206 -1.338514 0.260245
O 5.830760 -1.857631 0.535934
Cu 4.032987 -1.106646 0.608971
O 2.110978 -0.832602 0.906207
C 1.536537 -1.015231 2.060181
O 0.311775 -0.957196 2.276089
Cu -1.397354 -0.622298 1.006954
O -1.813340 -2.493122 1.252150
C -2.958251 -2.990777 1.012855
O -3.945321 -2.487989 0.395716
O 5.844872 1.418057 1.865526
C 5.167607 0.664407 2.628326
O 4.434707 -0.335408 2.346259
O 3.575584 -2.628130 -0.541025
C 2.649180 -2.834968 -1.378718
O 1.698581 -2.075581 -1.756799
O 0.693791 1.449910 -0.649942
C -0.228755 1.886361 0.108301
O -1.063078 1.268581 0.839009
O -2.752861 -0.119027 2.371908
C -3.896050 0.291973 2.042270
O -4.465805 0.201053 0.896651
H 6.620256 4.179029 -1.998958
H 7.814093 -2.045906 0.271120
H 5.224182 0.915437 3.710056
H 4.292823 1.430201 -2.615939

H 2.663254 -3.835167 -1.867020
H 2.228968 -1.232598 2.900748
H -0.316124 2.994897 0.139525
H -0.621430 -1.956960 -2.599670
H -4.495087 0.795214 2.826869
H -3.113674 -4.019651 1.400266
H -7.417719 -1.311093 -2.428089
Cu -6.170700 1.308169 0.040868
O -3.268576 0.749051 -1.607150
C -3.950432 1.808988 -1.779255
O -5.083198 2.140407 -1.315666
H -3.474067 2.559830 -2.447763
O -7.672952 1.385571 1.403476
O -5.957643 2.788313 1.459253
C -7.062136 2.369262 1.939068
H -7.499846 2.866607 2.830738

Cu8(HCO2)17-
(DF-PBE/6-31G*)
E=-16333.031970
C -6.035635 3.148327 -3.430000
H -6.173839 3.276539 -4.523976
O -4.593240 2.026976 1.788655
O -4.874470 3.250637 -2.916759
O -7.053066 2.906070 -2.695632
O -7.290281 1.641785 1.618462
C -7.686507 2.732733 1.108431
H -8.552579 3.208893 1.618000
O -7.244275 3.364260 0.097852
O -4.431838 3.502380 0.037129
C -4.134987 3.048186 1.184390
H -3.368718 3.625823 1.743662
Cu -5.721346 0.601470 1.108281
O -5.243165 0.771706 -1.025130
O -4.127817 -0.603767 1.332347
H -4.002577 0.916207 -2.631689
C -4.308455 0.310325 -1.752667
O -3.665583 -0.777328 -1.597353
Cu -3.913773 -2.082056 -0.219458
O -5.921303 -2.308236 -0.401751
C -6.857936 -1.921240 0.347024
H -7.781510 -2.545753 0.352253
O -6.922551 -0.901310 1.116455
O -3.940802 -3.494998 1.094579
C -2.873710 -3.966765 1.598394
O -2.666989 -3.215397 -1.315811
H -2.989396 -4.934704 2.129466
O -1.695021 -3.495253 1.580996
C -1.453041 -2.948948 -1.463605
H -0.898729 -3.427015 -2.292715
O -0.754108 -2.155427 -0.717924
Cu -1.310616 -1.653390 1.119768
O -2.367412 -1.073967 2.682423
C -3.492223 -0.560511 2.451202
H -3.985679 -0.027955 3.286303
O -1.072455 0.217985 0.703425
C -0.255681 0.756994 -0.106192
H -0.338745 1.862515 -0.188153
Cu 1.160725 -1.591271 -1.110836
O 0.644908 0.237626 -0.839088
O 0.571355 -1.835116 2.212704
C 1.775774 -1.870626 1.909893
H 2.546468 -1.957118 2.704123
O 2.248792 -1.808679 0.694004
O 1.526360 -3.387331 -1.683266
C 2.586246 -4.038248 -1.398815
H 2.597141 -5.085504 -1.774997
O 3.616353 -3.673755 -0.762047
O 2.204714 -0.912815 -2.666880
C 3.334096 -0.384130 -2.496887
H 3.762624 0.187042 -3.344874
O 4.036112 -0.456280 -1.420297
O 4.714885 -1.235339 1.876226
Cu 4.123490 -2.040193 0.212852
Cu 5.674544 0.636356 -1.254976
C 5.241560 -0.078741 1.925288
H 5.539386 0.271896 2.936727
O 5.460749 0.701400 0.947310
O 5.940727 -2.574226 -0.185974
C 6.772122 -2.070633 -1.002466
H 7.640626 -2.722563 -1.247613
O 6.771323 -0.939302 -1.579444
Cu -5.833716 2.854458 -1.102902
Cu 5.836772 2.819998 1.150229
O 7.338010 1.617607 -1.513138
O 7.541943 3.024913 0.295690
C 7.919293 2.482981 -0.789542
O 4.670775 2.152972 -1.920043
O 4.862476 3.714773 -0.245314
C 4.478313 3.285856 -1.374910
H 3.885922 4.005269 -1.979970
H 8.911750 2.818534 -1.162727
O 6.573286 2.775333 3.072541
O 4.508646 3.392293 2.564401
C 5.422583 3.194546 3.432284
H 5.214338 3.389922 4.504645
